# PYRIDOX(AM)INE 5′-PHOSPHATE OXIDASE3 of *Arabidopsis thaliana* maintains carbon/nitrogen balance in distinct environmental conditions

**DOI:** 10.1093/plphys/kiad411

**Published:** 2023-07-15

**Authors:** Priscille Steensma, Marion Eisenhut, Maite Colinas, Laise Rosado-Souza, Alisdair R Fernie, Andreas P M Weber, Teresa B Fitzpatrick

**Affiliations:** Department of Plant Sciences, University of Geneva, Geneva 1211, Switzerland; Institute of Plant Biochemistry, Cluster of Excellence on Plant Science, Heinrich-Heine-University, Düsseldorf 40225, Germany; Department of Plant Sciences, University of Geneva, Geneva 1211, Switzerland; Max-Planck-Institut für Molekulare Pflanzenphysiologie, Potsdam-Golm 14476, Germany; Max-Planck-Institut für Molekulare Pflanzenphysiologie, Potsdam-Golm 14476, Germany; Institute of Plant Biochemistry, Cluster of Excellence on Plant Science, Heinrich-Heine-University, Düsseldorf 40225, Germany; Department of Plant Sciences, University of Geneva, Geneva 1211, Switzerland

## Abstract

The identification of factors that regulate C/N utilization in plants can make a substantial contribution to optimization of plant health. Here, we explored the contribution of pyridox(am)ine 5′-phosphate oxidase3 (PDX3), which regulates vitamin B_6_ homeostasis, in *Arabidopsis* (*Arabidopsis thaliana*). Firstly, N fertilization regimes showed that ammonium application rescues the leaf morphological phenotype of *pdx3* mutant lines but masks the metabolite perturbance resulting from impairment in utilizing soil nitrate as a source of N. Without fertilization, *pdx3* lines suffered a C/N imbalance and accumulated nitrogenous compounds. Surprisingly, exploration of photorespiration as a source of endogenous N driving this metabolic imbalance, by incubation under high CO_2_, further exacerbated the *pdx3* growth phenotype. Interestingly, the amino acid serine, critical for growth and N management, alleviated the growth phenotype of *pdx3* plants under high CO_2_, likely due to the requirement of pyridoxal 5′-phosphate for the phosphorylated pathway of serine biosynthesis under this condition. Triggering of thermomorphogenesis by growth of plants at 28 °C (instead of 22 °C) did not appear to require PDX3 function, and we observed that the consequent drive toward C metabolism counters the C/N imbalance in *pdx3*. Further, *pdx3* lines suffered a salicylic acid-induced defense response, probing of which unraveled that it is a protective strategy mediated by nonexpressor of pathogenesis related1 (NPR1) and improves fitness. Overall, the study demonstrates the importance of vitamin B_6_ homeostasis as managed by the salvage pathway enzyme PDX3 to growth in diverse environments with varying nutrient availability and insight into how plants reprogram their metabolism under such conditions.

## Teaser

The contribution of pyridox(am)ine 5′-phosphate oxidase3 (PDX3) to vitamin B6 homeostasis is an important component of C/N balance in *Arabidopsis* grown in nitrate and high CO_2_, during which it assists serine biosynthesis.

## Introduction

Vitamin B_6_ in the form of the vitamer pyridoxal-5′-phosphate (PLP) is an important coenzyme for numerous enzymes mainly comprising amino acid biosynthesis (Liu Z et al. 2022). Additional functions have been attributed to vitamin B_6_ pertaining to transcription, facilitating protein folding or contributing to antioxidant activity (Liu Z et al. 2022). While vitamin B_6_ biosynthesis de novo is well characterized in plants, notably *Arabidopsis* (*Arabidopsis thaliana*) (Tambasco-Studart et al. 2005, 2007; Robinson et al. 2016), salvage pathways of vitamin B_6_ have also received increasing attention in the past years and revealed their essentiality for plant development and coping with environmental stresses (Shi et al. 2002; Shi and Zhu 2002; González et al. 2007; Herrero et al. 2011; Colinas et al. 2016; Colinas and Fitzpatrick 2016; Gorelova et al. 2022). Three salvage pathway enzymes have been identified so far, namely, a PYRIDOXAL (PL) KINASE (SOS4) (Shi et al. 2002; Shi and Zhu 2002), a PL REDUCTASE (PLR1) (Herrero et al. 2011) and a PYRIDOXINE 5′-PHOSPHATE (PNP)/PYRIDOXAMINE 5′-PHOSPHATE (PMP) oxidase (PYRIDOX(AM)INE 5′-PHOSPHATE OXIDASE3, PDX3) (Sang et al. 2007). Here, we focus on the latter and in *Arabidopsis* (At5g49970). In the absence of PDX3 in *Arabidopsis*, there is accumulation of the PNP and PMP forms of vitamin B_6_ and a deficit in PLP, consistent with its biochemical function (Colinas et al. 2016). Notably though, PDX3 is a 2-domain enzyme, 1 of which is assigned the PMP/PNP oxidase activity (POX domain), and the other harbors an epimerase activity associated to a pathway for repair of hydrated forms of NAD(P)H (NNRE domain) (Colinas et al. 2014; Niehaus et al. 2014). Although, more recent evidence suggests that the physiological function of the NNRE domain is rather associated with vitamin B_6_, as inactivation resulted in disruption of PLP homeostasis (Niehaus et al. 2018). The POX domain has also been reported to oxidize 6-NAD(P)H to NAD(P)^+^; however, the specificity constant is much lower than for PNP or PMP (Marbaix et al. 2019). Moreover, a different *Arabidopsis* protein (At2g46580) was shown to specifically oxidize 6-NAD(P)H in the same study (Marbaix et al. 2019) and shows very low percentage identity (8%) to PDX3 (Supplemental Fig. S1). In *Arabidopsis*, removal of PDX3 is coincident with an alteration of metabolite levels, in particular a bias toward accumulation of nitrogenous compounds, not seen in other vitamin B_6_ metabolism mutants, and a strong morphological phenotype with reduced leaf and seed biomass (Colinas et al. 2016). A conundrum is the ability to rescue the *pdx3* phenotype by provision of nitrogen (N) in the form of exogenous ammonium (but not nitrate) despite the already increased N load in these lines (Colinas et al. 2016). Intriguingly, nitrate assimilation is impaired in *pdx3* mutants due to decreased nitrate reductase (NR) activity. Furthermore, a constitutive defense response is evident in *pdx3* lines in the absence of pathogen challenge (autoimmunity) that is coincident with an accumulation of salicylic acid (SA) (Colinas et al. 2016). Whether SA contributes to the growth defects in *pdx3* has not been explored. Moreover, the connection between vitamin B_6_ balance, N metabolism, and SA-induced defense is unknown but could provide crucial information on the importance of this vitamin for metabolic homeostasis in plants under particular environmental challenges.

A fundamental aspect of cellular homeostasis for autotrophic organisms is maintenance of carbon (C)/N balance vital for interconnecting CO_2_ fixation with N assimilation (Nunes-Nesi et al. 2010). Plants source N exogenously from their surrounding environment or endogenously through recycling of nitrogenous compounds (e.g. amino acids) or from photorespiration in the case of C_3_ plants. Under aerobic and slightly acidic conditions, the presence of nitrifying bacteria in soil leads to the oxidation of ammonium to nitrate via nitrite; thus nitrite and ammonium levels are usually low (Chalk and Smith 2021; Subbarao and Searchinger 2021). A considerable portion of crop cultivation of nonlegumes is carried out on soil that is under such conditions and invokes the high energy-consuming nitrate and nitrite reductase reactions of the plant to reduce nitrate back to ammonium for assimilation into nitrogenous compounds required by the plant for growth. Nonetheless, the paucity of N sources in current soils and the drive since decades to increase plant yields, which has greatly benefitted food security, has led to vast amounts of synthetic fertilizer being applied routinely to replenish N. However, as plants only absorb a fraction of the fertilizer applied, run-off has led to environmental pollution negatively impacting ecosystems and biodiversity (Tilman et al. 2011; Poore and Nemecek 2018). Rising CO_2_ levels compound the problem because nitrate uptake is believed to be compromised under high CO_2_ (Bloom et al. 2010, 2020). Therefore, to improve N use efficiency, knowledge of mechanisms that regulate N management by plants is critical.

We explored the contribution of PDX3 to N management by performing metabolite analyses on Arabidopsis *pdx3* lines upon N fertilization. While ammonium application rescues the morphological phenotype of *pdx3* mutants, it masks the metabolite perturbance resulting from the impairment in these lines in utilizing soil nitrate as a source of N. Indeed, our data suggest that *pdx3* has little effect on the metabolism that operates in the presence of exogenous ammonium. In the absence of fertilization, *pdx3* mutants suffer a C/N imbalance and accumulate nitrogenous compounds. Surprisingly, exploration of photorespiration as a source of endogenous N driving this metabolic imbalance, by incubation under high CO_2_, further exacerbated the *pdx3* growth phenotype. Interestingly, the amino acid serine, which is critical for growth and N management (Zimmermann et al. 2021), alleviates the growth phenotype of *pdx3* plants under high CO_2_. Triggering of thermomorphogenesis by growth of plants at 28 °C (instead of 22 °C) is another condition that does not appear to require PDX3 function, and we observed that the consequent drive toward C metabolism counters the C/N imbalance in *pdx3*. Further probing of the SA-induced defense response in *pdx3* led us to unravel that it is a protective strategy mediated by NPR1 and improves fitness. Overall, the study demonstrates the importance of vitamin B_6_ homeostasis as managed by the salvage pathway enzyme PDX3 to growth in diverse environments with varying nutrient availability and insight into how plants reprogram their metabolism under such conditions.

## Results

### Metabolism of exogenous ammonium is not reliant on *PDX3*

**Figure 1.**
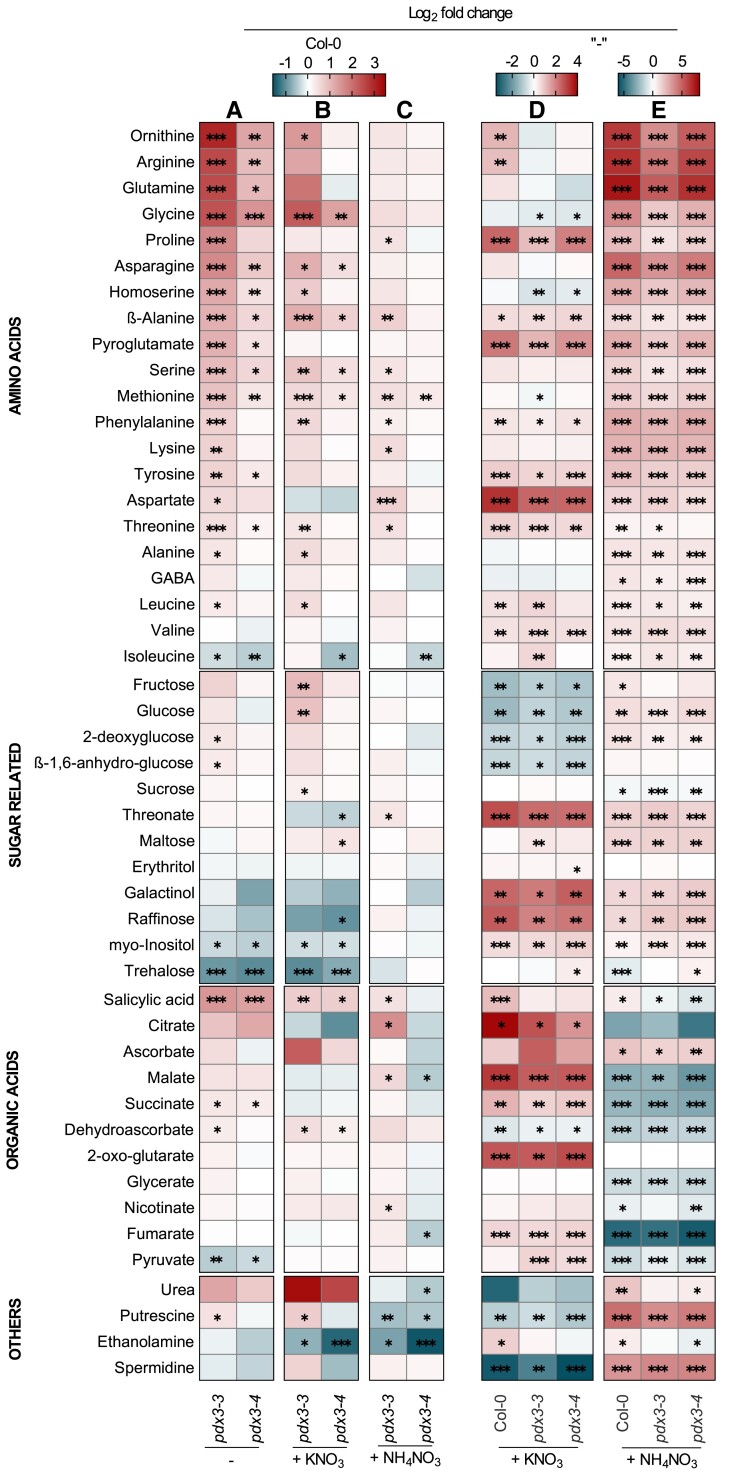
Metabolite profiles of *pdx3* plants compared to wild type under nitrogen fertilization regimes. Relative metabolite contents of *pdx3* lines are presented as heatmaps compared to wild type (Col-0) either without fertilization (−) **A)**, or with potassium nitrate fertilization (+KNO_3_) **B)**, or with ammonium nitrate fertilization (+NH_4_NO_3_) **C)** (i.e. ratio of contents in *pdx3* compared to Col-0, both under each condition). An analysis of the same set of data in (**A** to **C**) is also presented in comparison to the metabolite profile of each respective line grown in the unfertilized condition (−). **D)** compares the unfertilized condition with that of potassium nitrate fertilization (+KNO_3_), and **E)** compares the unfertilized condition with that of ammonium nitrate fertilization (+NH_4_NO_3_). The data are represented as the Log_2_ of the average fold change (*n* = 5 to 6) either to wild type (Col-0) or the condition (−). Statistical analysis was performed using a 2-tailed Student's unpaired *t*-test for *P ≤ 0.05, **P ≤ 0.005, and ***P ≤ 0.0005 on fold change results using line Col-0 or condition (−) as control. The analysis was performed on 21-d-old rosettes of plants grown on soil under a 16-h photoperiod (120 to 190 *µ*mol photons m^−2^ s^−1^) at 22 °C and 8-h darkness at 18 °C. Plants were watered with water alone (−) or a 50 mM solution of the indicated compound every 9 to 10 d.

Our previous analyses on *pdx3* plants (*pdx3-3*/*pdx3-4*, strong and weak allele, respectively) revealed a considerable alteration in the metabolite profile compared to wild type (Colinas et al. 2016). In particular, there is a strong accumulation of nitrogenous compounds in these *pdx3* plants grown on soil in contrast to wild type. Here, in an effort to dissect the source of metabolite disturbance, we compared the metabolite profiles of soil-grown *pdx3* lines and wild-type plants in the absence and presence of fertilization with different N sources. In the absence of any fertilization, the profile of the *pdx3* lines was different to that of wild type with a strong enrichment of nitrogenous compounds including several amino acids and urea, corroborating previous observations (Colinas et al. 2016) (Fig. 1A). There were consistent significant decreases in myoinositol, trehalose, and pyruvate, although succinate was increased (Fig. 1A). In the presence of potassium nitrate fertilization, there were significant metabolite changes in *pdx3* plants compared to the wild type, many of which were similar to those under the unfertilized conditions (Fig. 1B). By contrast, supplementation with ammonium nitrate dampened the metabolite profile divergence in *pdx3* plants and was very similar to that of wild type under the same conditions (Fig. 1C). A comparison of wild type under the unfertilized condition to that of all lines under the fertilization conditions indicated that the changes in metabolism of wildtype and *pdx3* plants upon potassium nitrate fertilization were divergent notably in relation to certain N-rich amino acids (Fig. 1D). On the other hand, the similar behavior of *pdx3* and wildtype plants upon ammonium nitrate fertilization was particularly striking (Fig. 1E). This observation suggested firstly that *pdx3* plants metabolize exogenous ammonium similar to wild type and secondly the divergent metabolite profile of *pdx3* lines in the unfertilized condition is largely masked upon addition of ammonium. Notably, exogenous ammonium supply rescues the narrow, elongated, thin leaves observed with *pdx3* plants grown on soil, whereas nitrate does not (Supplemental Fig. S2), as observed previously (Colinas et al. 2016). Nonetheless, the strong accumulation of nitrogenous compounds seen upon ammonium fertilization is what is observed for *pdx3* plants in the absence of fertilization, particularly the enhancement of amino acids (Fig. 1, A and E).

**Figure 2.**
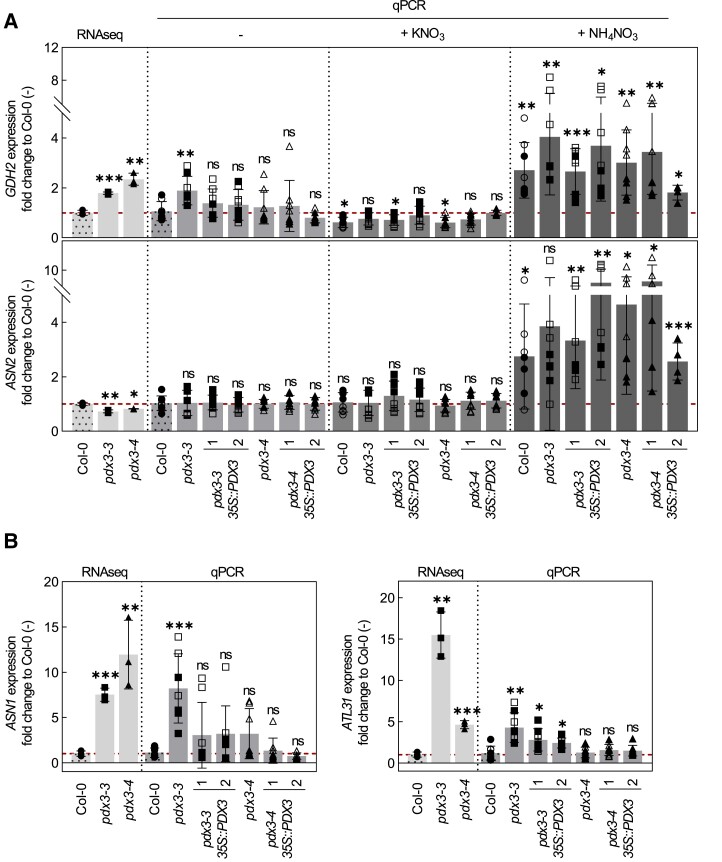
Marker gene evidence for C/N imbalance in *pdx3*. **A)** Relative expression fold change, as determined by RNA-seq (1) or RT-qPCR (qPCR), of *GDH2* (marker for both endogenous and exogenous ammonium assimilation) and *ASN2* (marker for exogenous ammonium assimilation) in wild type (Col-0), *pdx3* and complementing lines grown either on unfertilized (−), potassium nitrate (+KNO_3_), or ammonium nitrate (+NH_4_NO_3_) fertilized soil compared to wild type grown on unfertilized soil. **B)***ASN1* (marker for C starvation and C/N imbalance) and *ATL31* (marker for C and N imbalance) in *pdx3* and complementing lines compared to wild type (Col-0). The data represent the mean ± Sd across 2 experimental replicates (either open or filled symbols) and with 4 biological replicates each. Statistical analysis was performed using a 2-tailed Student's unpaired *t*-test with unfertilized Col-0 as control (^ns^*P* > 0.05, *P ≤ 0.05, **P ≤ 0.005, and ***P ≤ 0.0005). The analysis was performed on 21-d-old rosettes of plants grown on soil under a 16-h photoperiod (120 to 190 *µ*mol photons m^−2^ s^−1^) at 22 °C and 8-h darkness at 18 °C. Either water alone (−) or a 50 mM solution of the indicated compound was supplemented to the soil every 9 to 10 d. The control was set to 1 and is indicated by the red dashed lines.

Given the strong bias toward enhanced N compounds in *pdx3* plants in the unfertilized condition, we checked molecular markers of N assimilation versus a response to exogenous ammonium via reverse transcription quantitative PCR (RT-qPCR). *ASPARAGINE SYNTHASE 2* (*ASN2*) is induced by exogenous ammonium (Wong et al. 2004), whereas *GLUTAMATE DEHYDROGENASE 2* (*GDH2*) induction marks ammonium assimilation (Patterson et al. 2010). Under standard growth conditions (in the absence of fertilization), *GDH2* was modestly induced in *pdx3* lines (particularly in the *pdx3-3* line), whereas *ASN2* expression was not significantly different to that in the wild type (Fig. 2A). This was supported by mining our previous RNA-seq data from these lines grown on soil (Colinas et al. 2016), which also showed that *GDH2* was induced in plants containing *pdx3* alleles compared to the wild type, whereas there was no difference in the *ASN2* levels (Fig. 2A). Importantly, *GDH2* was not induced in *pdx3* lines carrying the *PDX3* transgene (complementing lines) in the absence of fertilization (Fig. 2A). On the other hand, both *GDH2* and *ASN2* were induced upon ammonium nitrate fertilization in all lines but not upon potassium nitrate fertilization (Fig. 2A). The aerobic conditions and slightly acidic soil used in this study are likely to contain no or very low levels of ammonium, as is also reflected by the absence of rescue of the ammonium-dependent leaf phenotype of *pdx3* mutants on unfertilized soil (Supplemental Fig. S1). This lent support to a notion that there may be a C/N imbalance in *pdx3* plants that imparts the morphological defect. Markers of C/N imbalance include induction of *ASPARAGINE SYNTHASE 1* (*ASN1*) or *ARABIDOPSIS TOXICOS EN LEVADURA 31* (*ATL31*) (Lam et al. 1994; Sato et al. 2009; Xu et al. 2019). Both were upregulated in *pdx3* plants compared to the wild type based on mining of the previous RNA-seq data and was supported by RT-qPCR of standard soil-grown samples in the absence of fertilization, notably in the *pdx3-3* line (Fig. 2B).

Taking all of the findings together, we infer that *PDX3* has little effect on the metabolism that operates in the presence of exogenous ammonium, and, thus, the requirement for *PDX3* is bypassed by ammonium fertilization. As exogenous ammonium metabolism appears to be intact in the *pdx3* lines, this then masks the metabolite disturbance and consequential morphological phenotype of these mutants observed in the unfertilized soil conditions, where nitrate is the predominant source of N.

### C/N imbalance in *pdx3* mutant plants is associated with the POX domain

**Figure 3.**
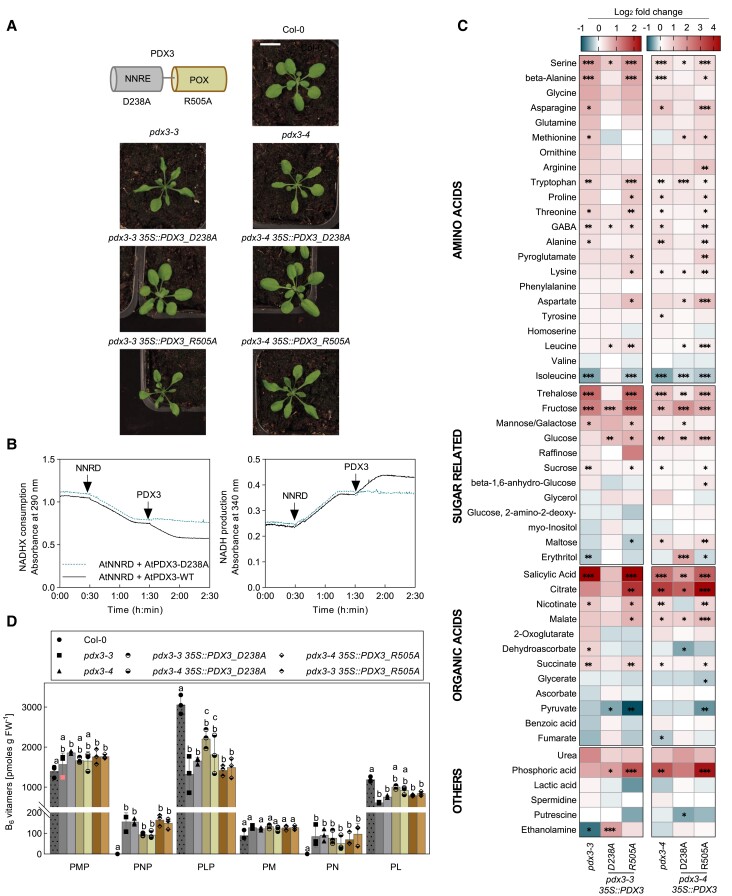
Contribution of the individual domains of PDX3 to the leaf phenotype and metabolite profile of *Arabidopsis*. **A)** Scheme of the 2 domains (NNRE and POX) of PDX3 (top left) and photographs of 21-d-old wild type (Col-0), *pdx3*, and lines expressing the *PDX3* transgene harboring either a D238A mutation in the NNRE domain or a R505A mutation in the POX domain. The scale bar applies to all photographs. Individual images were digitally extracted for comparison. **B)** NADHX repair assay. (*S*)-NADHX within a racemic mixture of NADHX is dehydrated to NADH by NNRD and followed by a decrease in absorption at 290 nm (left panel) or increase at 340 nm (right panel). Subsequent addition of PDX3 wild type (black trace) leads to epimerization of (*R*)-NADHX in the racemate to (*S*)-NADHX by the NNRE domain, providing further substrate for NNRD. PDX3 D238A (blue dotted trace) cannot carry out the epimerase reaction. **C)** Relative metabolite contents of 14-d-old *pdx3* and lines carrying the PDX3 D238A or PDX3 R505A transgene are presented as heatmaps compared to Col-0. The data are represented as the Log_2_ of the average fold change (*n* = 5 to 6) to Col-0. Statistical analysis was performed using a 2-tailed Student's unpaired *t*-test for *P ≤ 0.05, **P ≤ 0.005, and ***P ≤ 0.005 on fold change results using Col-0 as control. **D)** Vitamin B_6_ profile (PMP, PNP, PLP, PM, PN, and PL) of 21-d-old rosettes of plants as indicated grown on soil (unfertilized) under a 16-h photoperiod (120 to 160 *µ*mol photons m^−2^ s^−1^) at 22 °C and 8-h darkness at 18 °C. The data represent the mean ± Sd of 3 biological replicates. Statistical analysis was performed using 2-way ANOVA with Tukey's multiple comparisons test (different letters indicate *P* ≤ 0.05).

Given that PDX3 harbors 2 domains with functionalities related to vitamin B_6_ homeostasis as well as repair of NAD(P)H, we next tested if both domains are required for C/N homeostasis. To this end, we mutated key active site residues in each domain of PDX3 (Supplemental Fig. S1) and introduced the modified sequences into *pdx3* mutants. In particular, residues D238 (NNRE domain) (Shumilin et al. 2012; Niehaus et al. 2018) and R505 (POX domain) (Di Salvo et al. 2003; Barile et al. 2020) were exchanged to alanines (Figs. 3A and S1). Interestingly, the morphology of *pdx3* lines carrying PDX3 D238A could not be distinguished from that of wildtype plants, whereas *pdx3* lines carrying PDX3 R505A resembled the *pdx3* mutant (Fig. 3A). Immunochemical analyses confirmed expression of the corresponding proteins (Supplemental Fig. S1). Moreover, the PDX3 D238A mutation was shown to abolish the epimerase activity of the NNRE domain in its function of repair of NAD(P)HX (Fig. 3B). We also determined the metabolite profile of all lines and noted that the perturbance in *pdx3* mutants was alleviated upon introduction of the PDX3 D238A mutant (Fig. 3C). By contrast, the metabolite profile of *pdx3* lines expressing the PDX3 R505A mutant was largely similar to that of the *pdx3* mutant itself (Fig. 3C). Previously, we have also shown that the loss of PDX3 leads to an imbalance in vitamin B_6_ levels, marked by an increase in PNP and PMP, as well as a decrease in PLP (and PL), in line with the biochemical activity of PDX3 (Colinas et al. 2016). Thus, we also determined the vitamin B_6_ profile of the lines generated here. We observed a slight decrease in PMP/PNP and increase in PLP (and PL) levels in *pdx3* lines expressing PDX3 D238A, thus countering the imbalance in the *pdx3* mutant (Fig. 3D). By contrast, the vitamin B_6_ profile of *pdx3* lines expressing PDX3 R505A was not statistically different to that of the *pdx3* lines (Fig. 3D).

This strongly suggests that both the morphological and metabolite perturbance of *pdx3* mutants is related to the POX domain (and its biochemical activity in vitamin B_6_ homeostasis) and not to the NNRE domain. The physiological relevance of the NNRE domain thus remains elusive but was not explored further in the context of this study.

### B_6_ vitamer alteration in *pdx3* plants does not directly account for reduced NR activity

**Figure 4.**
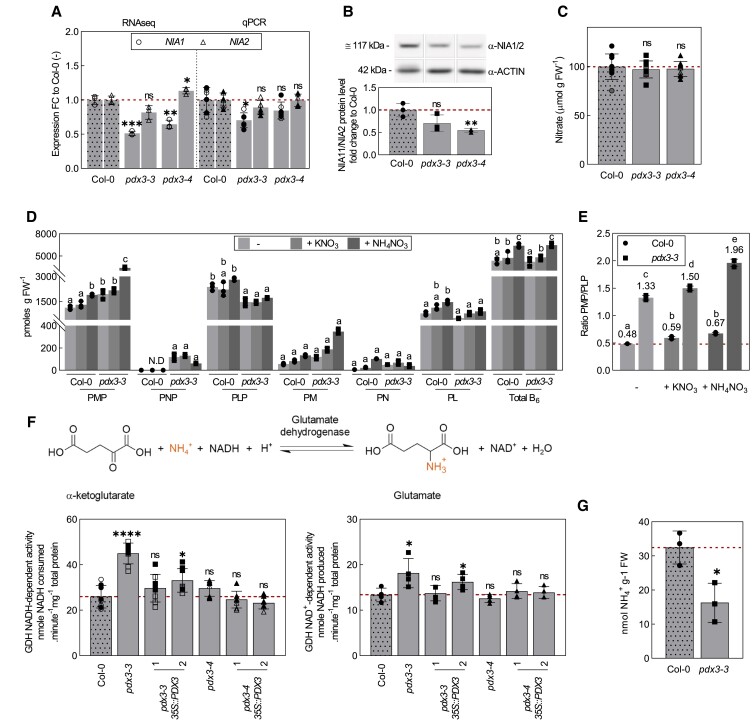
Alteration of vitamin B_6_ homeostasis does not directly impact NR activity. **A)** Relative expression fold change in expression of *NIA1* and *NIA2* in *pdx3* compared to wild type (Col-0), as determined by RNA-seq (1) and by RT-qPCR (qPCR). **B)** NIA1/2 protein levels of lines as in (A), determined by immunochemistry using 5 mg of total protein. **C)** Nitrate content of lines as in (A). **D)** Vitamin B_6_ profile of *pdx3-3* and wildtype (Col-0) plants grown on unfertilized (−), potassium nitrate (+KNO_3_) or ammonium nitrate (+NH_4_NO_3_) fertilized soil. **E)** PMP/PLP ratios in wild type and *pdx3-3* from the data presented in (D). **F)** GDH activity in *pdx3* compared to wild type (Col-0). The top panel shows a scheme of the reaction; the bottom left panel shows NADH-dependent GDH activity and bottom right NAD^+^-dependent GDH activity of plants. **G)** Free ammonium levels in *pdx3-3* compared to wild type (Col-0). Expression data in **A)** and **B)** represent the mean ± Sd across 1 to 2 experimental replicates (either open or filled symbols) with 3 biological replicates each. The data in **C)** represent the mean ± Sd across 2 experimental replicates (filled, open, or semi-filled symbols) with 3 to 6 biological replicates each. The data in **D)** and **E)** represent the mean ± Sd of 3 biological replicates. The data in **F)** represent the mean ± Sd across 1 to 2 experimental replicates (either open or filled symbols) with 4 biological replicates each. The data in **G)** represent the mean ± Sd across 2 to 3 biological replicates. For **A)** to **C)** and **F)** to **G)**, statistical analyses were performed using a 2-tailed Student's unpaired *t*-test with unfertilized Col-0 as control (^ns^*P* > 0.05 and *P ≤ 0.05, **P ≤ 0.005 ***P ≤ 0.0005, and ****P ≤ 0.00005). For **D)** to **E)**, statistical analyses were performed using a 2-way ANOVA with a Tukey's multiple comparison test. Different letters indicate *P* ≤ 0.05 within each vitamer sub-categories in **D)**, whereas in **E)**, different letters indicate *P* ≤ 0.05 across all categories. The dashed line in **A)** and **B)** indicates the control set to 1 in **A)** and **B)** or the wild type (Col-0) in **C)** to **G)**. The analyses in **A)** to **F)** were performed on 21-d-old rosettes of plants grown on soil under a 16-h photoperiod (120 to 190 *µ*mol photons m^−2^ s^−1^) at 22 °C and 8-h darkness at 18 °C. Either just water (**A** to **C**, F or “−”) or a 50 mM solution of the indicated compound was supplemented to the soil every 9 to 10 d. The analysis in **G)** was performed on 9-d-old rosettes of plants grown on plates with modified MS medium containing no ammonium under a 12-h photoperiod (120 *µ*mol photons m^−2^ s^−1^) at 22 °C and 12-h darkness at 18 °C.

In contrast to ammonium fertilization, nitrate does not rescue the *pdx3* morphological phenotype as mentioned above (Supplemental Fig. S2). This is not surprising because previous studies have shown that NR activity is reduced in these *pdx3* lines (Colinas et al. 2016), although it is not known why. Biosynthesis of the NR protein is inhibited by certain amino acids as well as when ammonium is used as a source of N assimilation (Huarancca Reyes et al. 2018; Kim et al. 2018). Thus, we measured transcript levels of *NIA1/2*, the 2 genes coding for NR in *Arabidopsis*, and noted that a significant reduction in *NIA1* in both *pdx3* lines from mining of the previous RNA-seq data set was corroborated by RT-qPCR for the *pdx3-3* line (Fig. 4A). Protein levels of NIA1/2 were also moderately reduced in both *pdx3* lines (Fig. 4B). However, nitrate levels were unchanged compared to wild type, suggesting that nitrate uptake is intact (Fig. 4C). We profiled the vitamin B_6_ content of *pdx3* lines versus wild type upon N fertilization and observed a marked increase in PMP when ammonium was applied irrespective of the genotype as before (Colinas et al. 2016) (Fig. 4D). Here, we also noted a significant (albeit modest) increase of PL in the wild type not seen in the *pdx3* lines, and although PNP was not detectable in wild type, it was clearly observed in *pdx3* lines (Fig. 4D). There were no other statistically significant changes in the individual vitamer profiles, but there was a general slight increase in vitamin B_6_ levels upon ammonium fertilization and no marked changes under nitrate fertilization (Fig. 4D). Notably, the PMP:PLP ratio in the *pdx3* lines remained much higher than that in wild type under all conditions (Fig. 4E). As it is well established that NR activity decreases upon ammonium fertilization (Huarancca Reyes et al. 2018; Kim et al. 2018), we tested if PMP could directly inhibit its activity. However, PMP at concentrations up to 2.5 mM, considerably higher than found in *Arabidopsis* tissue (Szydlowski et al. 2013), had only a mild impact on NR activity (Supplemental Fig. S3A). Thus, it is unlikely that PMP directly inhibits NR activity. Furthermore, NR activity is time of day regulated, peaking early in the day, which was maintained in *pdx3* plants, although a lower activity was observed throughout the day (Supplemental Fig. S3B). We also tested if N fertilization could affect *PDX3* expression levels. However, neither transcript nor protein levels changed under either ammonium or nitrate fertilization (Supplemental Fig. S3, C and D). Thus, it is rather the strong accumulation of nitrogenous compounds (amino acids) in *pdx3* plants that is likely to trigger negative feedback regulation on NR, as reported previously for wildtype plants fed with ammonium (Huarancca Reyes et al. 2018; Kim et al. 2018). As amino acids are increased in *pdx3* plants even without ammonium supply and *GDH* expression is induced, we measured GDH activity and noted that it was considerably increased in the direction of glutamate formation (ammonium consumption) in the *pdx3* lines, a condition that was reversed in the complementing lines (Fig. 4F). We also measured free ammonium levels and noted a significant decrease in *pdx3* lines compared to wild type (Fig. 4G). These data suggest that internal ammonium assimilation is enhanced in *pdx3* lines and could at least partially account for the elevated amino acid levels, although, decreased protein metabolism, or amino acid catabolism, could also contribute.

We infer that the biochemical function of PDX3 in maintaining PMP levels does not appear to be directly related to the reduced activity of NR in *pdx3* lines, rather the accumulation of amino acids (with a contribution from enhanced ammonium assimilation) likely negatively feedback on NR biosynthesis and explain the reduced transcript/protein/activity levels.

### Growth of *pdx3* lines further deteriorates under high CO_2_ and is alleviated by serine supplementation

**Figure 5.**
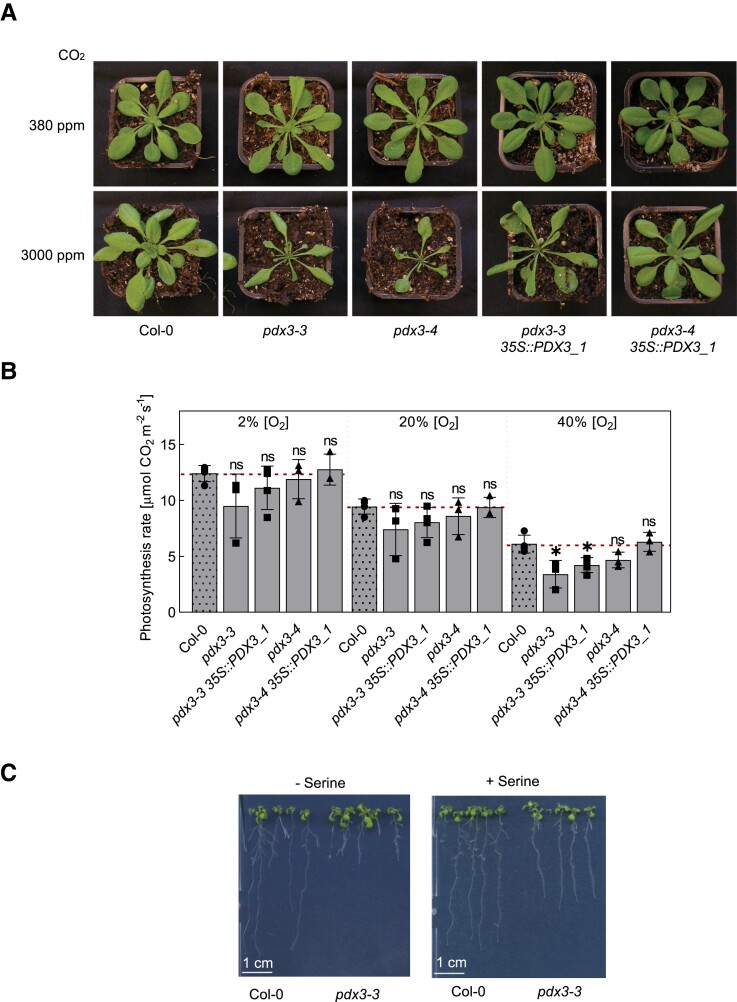
Growth of *pdx3* further deteriorates under high CO_2_ and is alleviated by serine supplementation. **A)** Phenotypic comparison of wild type (Col-0), *pdx3*, and complementing lines grown under elevated CO_2_ (3000 ppm) or ambient CO_2_ (380 ppm) on soil and a 12-h photoperiod (120 *µ*mol photons m^−2^ s^−1^) at 22 °C and 12 h of darkness at 18 °C. The scale bar applies to all photographs. Individual images were digitally extracted for comparison. **B)** Analysis of the rate of photosynthesis as a function of the oxygen percentage in *pdx3* and complementing lines compared to wild type (Col-0). Plants were grown under the same conditions as in **A)**. The data represent the mean ± Sd of 3 experimental replicates. Statistical analysis was performed using a multiple 2-tailed Student's unpaired *t*-test using the Holm–Sidak method with Col-0 as control (^ns^*P* > 0.05 and *P ≤ 0.05). **C)** Growth of wild type (Col-0) and *pdx3* on culture plates under elevated CO_2_ (3000 ppm) in the absence or presence of serine (100 *µ*M) supplemented to the medium. Individual images were digitally extracted for comparison.

Photorespiration in C_3_ plants such as *Arabidopsis* is an important metabolic route contributing to C/N balance (Eisenhut et al. 2019). We entertained a notion that as photorespiration constrains CO_2_ assimilation and is an important source of endogenous ammonium, its repression may alleviate the C/N imbalance in *pdx3* lines and improve growth. Metabolic flux through the photorespiratory cycle can be repressed by exposing plants to elevated CO_2_ during which oxygen used by Rubisco is diminished. However, exposure of *pdx3* lines to elevated CO_2_ further exacerbated the leaf phenotype of *pdx3* compared to that under ambient CO_2_ (Fig. 5A). This suggested that contrary to our original notion, photorespiration is beneficial to *pdx3* lines. Notwithstanding, the photosynthesis rate of *pdx3* lines was significantly impaired under high O_2_, a condition that favors photorespiration (Fig. 5B).

We remained puzzled by the bias toward enhanced nitrogenous compounds in *pdx3* lines and strived to define which cellular functions were perturbed by the lack of PDX3 activity. Recently, the phosphorylated pathway of serine biosynthesis (PPSB) has been shown to be vital for *Arabidopsis* plant growth (Zimmermann et al. 2021). The PPSB pathway is predominantly active in plant roots and is a major contributor to the serine pool, especially under elevated CO_2_, when photorespiration is repressed (Zimmermann et al. 2021). Mutations in the PPSB pathway lead to an accumulation of nitrogenous compounds and a severe impact on growth, particularly under high CO_2_ conditions (Zimmermann et al. 2021). The parallels with the phenotypes of *pdx3* allowed us to postulate that *pdx3* plants may suffer a deficiency in serine biosynthesis through the PPSB pathway. This notion was supported by the fact that 3-phosphoserine aminotransferase (PSAT), an enzyme of the PPSB pathway, is dependent on PLP for activity (Wulfert and Krueger 2018). As external feeding with serine has been shown to be metabolized the same way as the plant would by biosynthesis de novo (Zimmermann et al. 2021), we tested if serine supplementation could improve growth of *pdx3* plants under elevated CO_2_. Indeed, growth of *pdx3* plants on culture plates under elevated CO_2_ was considerably improved in the presence of serine (Fig. 5C).

This suggests that serine biosynthesis through the PPSB pathway is compromised in *pdx3* plants and contributes to the C/N imbalance in these lines. Moreover, our data clearly indicate that PDX3 function is crucial under elevated CO_2_ levels.

### A shift in metabolism at elevated temperatures compensates for loss of *PDX3*

**Figure 6.**
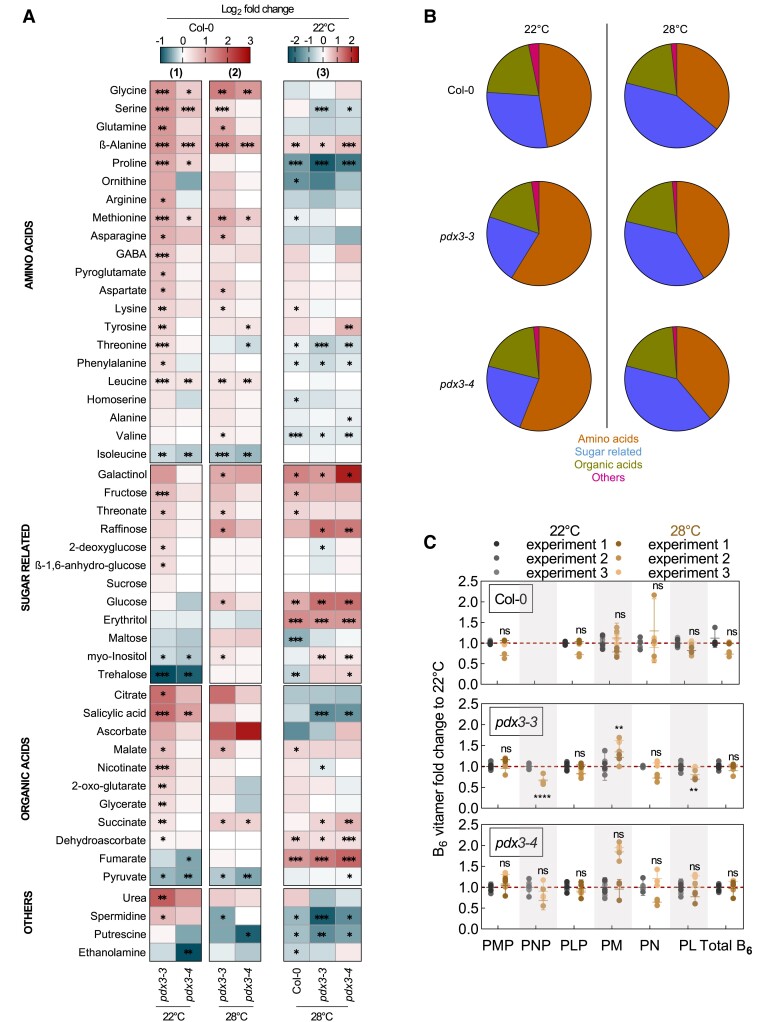
Metabolite profiling of wildtype and *pdx3* plants grown under different temperatures. **A)** Relative metabolite contents of *pdx3* lines are presented as heatmaps compared to wild type (Col-0) at 22 °C (panel 1), or of *pdx3* at 28 °C compared to 22 °C (Panel 2), or of wild type and *pdx3* compared to wild type at 22 °C (Panel 3). The data are represented as the Log_2_ of the average fold change (*n* = 5 to 6) to Col-0 or condition (22 °C). Statistical analysis was performed using a 2-tailed Student's unpaired *t*-test for *P ≤ 0.05, **P ≤ 0.005, and ***P ≤ 0.0005 on fold change results using line Col-0 or condition (22 °C) as control. **B)** Pie-chart representation of the proportions of relative abundance of each group of metabolites within the total amount of metabolites measured. **C)** Fold change in B_6_ vitamers of wildtype and *pdx3* lines grown at 28 °C (brown) compared to 22 °C (gray). The data represent the mean ± Sd across 3 independent experimental replicates (represented by different shades of brown and gray, respectively). Statistical analysis was performed using a 2-tailed Student's unpaired *t*-test using the individual vitamers at 22 °C as control (^ns^*P* > 0.05, **P ≤ 0.005, and ****P ≤ 0.0005). The control (vitamer at 22 °C) was set to 1 and is indicated by the red dashed line. The analysis was performed on rosettes of plants grown on soil (unfertilized) up to 14 DAG under a 16-h photoperiod (120 to 190 *µ*mol photons m^−2^ s^−1^) at 22 °C and 8-h darkness at 18 °C (22 °C) or up to 12 DAG at constant 28 °C (28 °C).

One other feature of *pdx3* lines is that they are morphologically similar to wildtype plants when grown at 28 °C (Colinas and Fitzpatrick 2016). In an effort to further probe the C/N imbalance in *pdx3* lines, we determined the metabolic status of lines grown at the standard 22 °C to that at 28 °C. As *Arabidopsis* plants proceed through developmental transitions faster at higher temperatures, we studied rosette leaves at either 14 or 12 d after germination (DAG) at 22 °C and 28 °C, respectively, time points at which 5 true leaves were present under both conditions (Supplemental Fig. S4). At 28 °C, there was a clear shift in the metabolic profile compared to at 22 °C such that the proportion of sugar-related compounds was higher in wild type as well as in the *pdx3* lines (Fig. 6, A and B). Thus, there was a reduction in the abundance of nitrogenous compounds in *pdx3* lines at the higher temperature, and the proportional distribution of metabolites was largely similar to that in the wild type at 28 °C. However, the vitamin B_6_ profile of *pdx3* mutant lines showed largely the same perturbations compared to that of the wild type at both temperatures (Fig. 6C). Notably, the vitamin B_6_ profile of wild type was not substantially altered at 28 °C compared to 22 °C (Fig. 6C).

The data indicate that there is a general shift in C/N balance at 28 °C with a higher proportion of C compounds and less investment in N compounds compared to that at 22 °C. The metabolic state at elevated temperature likely compensates the C/N imbalance observed in *pdx3* lines at 22 °C, and thus growth of *pdx3* plants matches that of wild type at 28 °C. These observations suggest metabolism at 28 °C is not reliant on PDX3 and interestingly is not noticeably affected by the imbalance of B_6_ vitamers, in contrast to metabolism at 22 °C.

### Molecular manipulation to decrease elevated SA levels in *pdx3* lines improves growth

**Figure 7.**
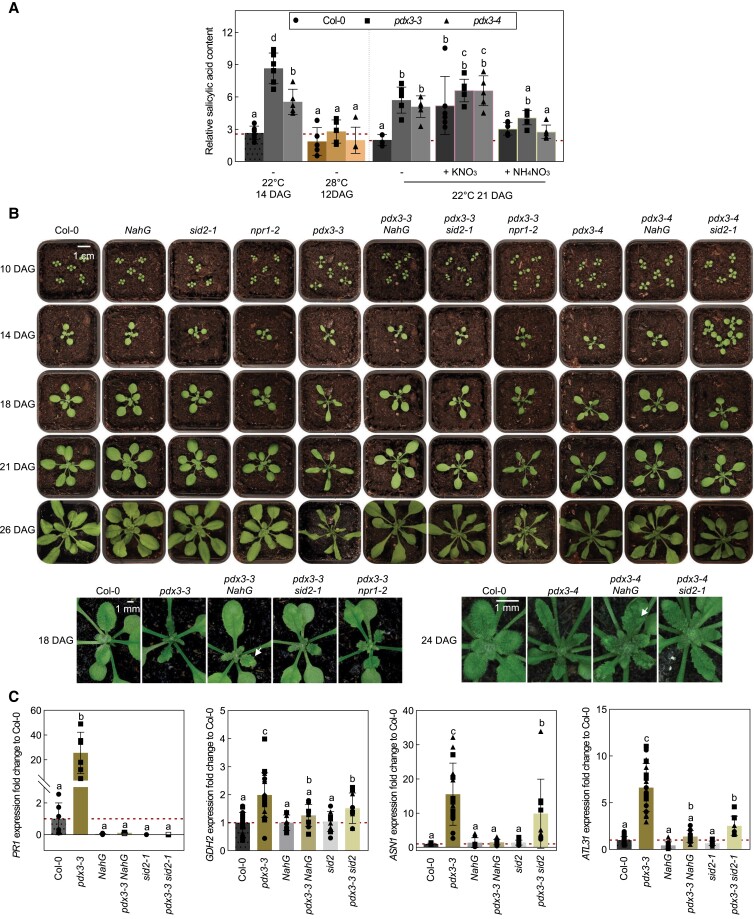
Contribution of SA to the *pdx3* leaf phenotype. **A)** Relative SA content in 12-, 14-, and 21-d-old wild-type (Col-0) and *pdx3* lines as indicated. Plants were grown on soil under either a 16-h photoperiod (120 to 190 *µ*mol photons m^−2^ s^−1^) at 22 °C and 8-h darkness at 18 °C (22 °C) or a 16-h photoperiod at 28 °C and 8-h darkness at 28 °C (28 °C). Either water alone (−) or a 50 mM solution of the indicated compound was supplemented to the soil every 9 to 10 d. The data represent the mean ± Sd of 5 to 6 biological replicates. **B)** Photographs of wild type (Col-0), *sid2-1*, *NahG*, *npr1-2*, and *pdx3* lines and *pdx3-3 sid2-1*, *pdx3-3 NahG*, *pdx3-3 npr1-2*, *pdx3-4 sid2-1*, and *pdx3-4 NahG* at various DAG as indicated. The lower left panel is a close-up of the corresponding lines shown in the upper panel. In the lower right panel are close-up photos of the lines as indicated 24 DAG. White arrows indicate leaf lesions. The scale bars apply to all photos grouped together. Individual images were digitally extracted for comparison. **C)** Relative expression fold change, as determined by RT-qPCR, of *PATHOGENESIS RELATED PROTEIN1* (*PR1*), GDH2, *ASN1,* and *Arabidopsis toxicos en levadura 31* (ATL31) in 21-d-old wild type (Col-0), *pdx3-3*, *NahG*, *pdx3-3 NahG*, *sid2-1*, and *pdx3-3 sid2-1*. The data represent the mean ± Sd across 3 independent experimental replicates (different squares, dots and triangles) of 3 to 6 biological replicates each. Statistical analysis in **A)** and **C)** was performed using a 1-way ANOVA with a Fisher's Lsd test (different letters indicate *P* ≤ 0.05). The level of the control (Col-0) in **A)** or the control set to 1 **C)** is indicated by the red dashed line. The analysis in **C)** was performed on rosette leaves of soil-grown plants (unfertilized) under a 16-h photoperiod (120 to 190 *µ*mol photons m^−2^ s^−1^) at 22 °C and 8-h darkness at 18 °C.

Another prominent feature of *pdx3* lines grown under standard conditions on soil is the elevation of transcripts related to the defense hormone SA, as well as SA itself (Colinas et al. 2016; Colinas and Fitzpatrick 2016). A central dogma in plant science is that growth is compromised when plants are in defense mode fending off attack (He et al. 2022). Interestingly, this trade-off can be alleviated by elevated temperatures and ammonium fertilization both of which reduce SA levels (Wang et al. 2013; Kim et al. 2021). In this context, we observed here that the SA content was considerably reduced in *pdx3* lines at 28 °C compared to 22 °C, as well as by fertilization with ammonium (Fig. 7A), i.e. conditions in which *pdx3* plants behave like wildtype plants. To dissect the contribution of SA to the phenotype of *pdx3* at 22 °C on unfertilized soil, we used a genetic approach to reduce SA levels. In particular, we crossed *pdx3* lines with the *SALICYLIC ACID DEFICIENT 2* mutant line (*sid2-1*) in which the isochorismate pathway of SA biosynthesis is blocked (Nawrath and Metraux 1999), as well as with the transgenic line carrying the *Pseudomonas putida NahG* gene that encodes salicylate hydroxylase and degrades SA into catechol (Lawton et al. 1995). While monitoring the growth of plants on soil throughout the vegetative stage of development, we noted that the leaf morphological defect in *pdx3* lines was already visible with the development of the first true leaves, which were narrower, were malformed in shape, and had a reduced leaf lamina area compared to those of the wild type (Fig. 7B). This feature was characteristic of all newly emerging leaves (Fig. 7B). On the other hand, these deformities were alleviated somewhat in the *pdx3-3 sid2-1*, *pdx3-4 sid2-1*, or *pdx3-3 NahG* or *pdx3-4 NahG* double mutant lines (Fig. 7B). In line with this, the expression of *PATHOGEN RESISTANT1* (*PR1*) was drastically reduced in these lines (Fig. 7C). However, we noted that although the leaf phenotype was improved, deformities in shape were still evident in developing leaves, and lesions were present that are not found in *pdx3* single mutants (Fig. 7B). Thus, while introgression of *sid2-1* or *NahG* improves *pdx3-3* and *pdx3-4* plant growth, it does not fully support a hypothesis where growth is compromised solely because of an SA-triggered defense response in *pdx3-3* and *pdx3-4* mutants. Interestingly, the increased transcript levels of *ASN1*, *ATL31*, and *GDH2* seen in *pdx3* single mutants, as markers of C/N imbalance, approached those of wild type in the *pdx3-3 NahG* and *pdx3-3 sid2-1* double mutants (Fig. 7C). Of note, these transcript levels were similar between wild type and the single *NahG* and *sid2-1* mutants at 22 °C (Fig. 7C). This suggested that SA likely contributes to C/N imbalance in *pdx3* lines. To probe this finding further, we crossed *pdx3-3* with *nonexpressor of PR genes 1-2* (*npr1-2*) (Cao et al. 1997) that is unable to transduce SA defense responses. Interestingly, the *pdx3-3 npr1-2* double mutant was not effective in alleviating the developmental defects of *pdx3-3* lines, in contrast to the *pdx3-3 sid2-1* or *pdx3-3 nahG* double mutant lines; indeed it appeared even worse (Fig. 7B). Thus, alternative pathways of SA biosynthesis that still operate in the *sid2-1* and *NahG* mutants, e.g. via phenylalanine ammonia lyase and are transduced by NPR1 (Nair et al. 2021), appear to benefit *pdx3* plant growth and will be interesting to dissect in future studies. Notably, the single mutant *sid2-1*, *nahG*, and *npr1-2* lines developed similar to wildtype plants under our conditions (Fig. 7B).

Taken together, the data suggest that while SA overaccumulation partially contributes to the developmental and morphological defects in *pdx3* mutants, it is not the primary cause of these abnormalities. Indeed, SA (likely at lower levels but needs to be confirmed) and its signaling via NPR1 prevent even poorer performance of *pdx3* lines.

### SA modulates vitamin B_6_ metabolism but cannot overcome a lack of PDX3 function

**Figure 8.**
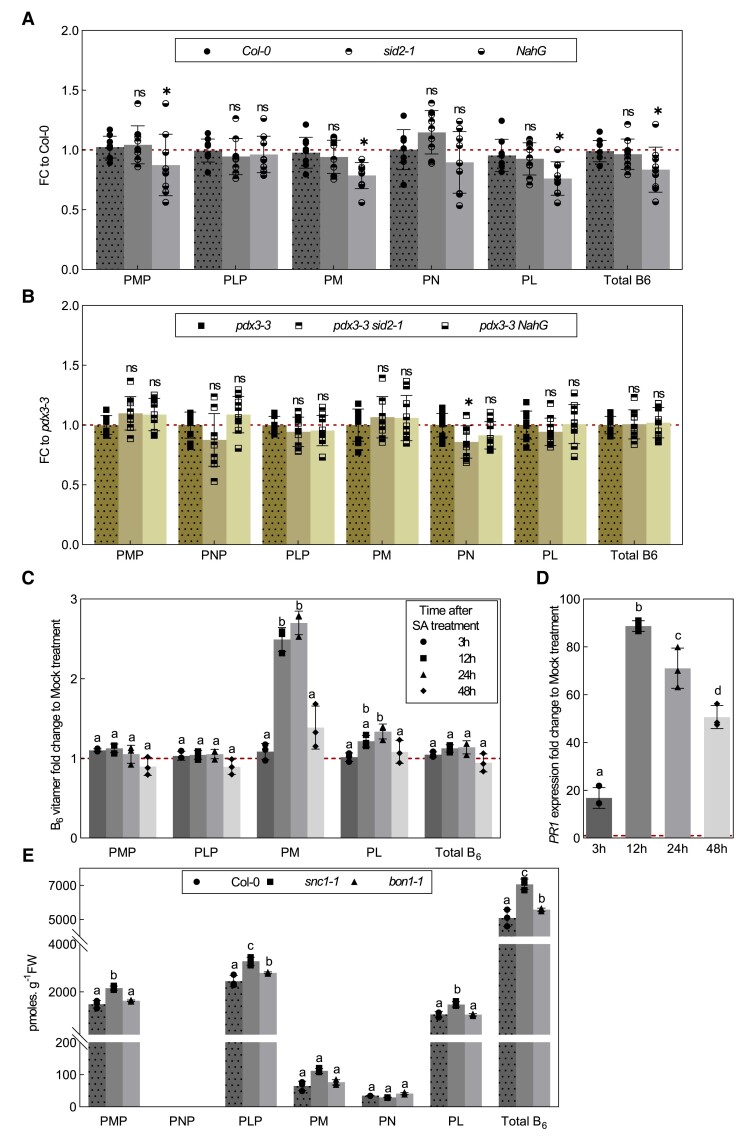
Salicylic acid can alter vitamin B_6_ levels. **A)** B_6_ vitamer content fold change of 21-d-old *sid2-1* and *NahG* plants normalized to the B_6_ vitamer content of wild type (Col-0) or **B)** of *pdx3-3 sid2-1* and *pdx3-3 NahG* normalized to the B_6_ vitamer content in *pdx3-3* alone. PMP, PNP, PLP, PM, PN, and PL. **C)** Fold change of the vitamin B_6_ profile of SA treated 19-d-old wild type (Col-0) at 0 h, 3 h, or 12 h after treatment, as well as 20 and 21 d old, at 24 and 48 h after treatment, respectively, where each time point was normalized to the corresponding mock-treated plant. **D)** Relative expression fold change (RT-qPCR) of *PATHOGENESIS RELATED PROTEIN1* (*PR1*) of SA-treated plants from **C)** at each time point compared to the respective mock treatment. **E)** Vitamin B_6_ profile and cumulative total vitamin B_6_ of 21-d-old wild type (Col-0) and the autoimmune mutants *snc1-1* (gain-of-function point mutation that leads to a constitutively active protein) and *bon1-1* (loss of function). The data in **A)** and **B)** represent the mean ± Sd across 3 independent experimental replicates of 3 biological replicates each. The data in **C)** to **E)** represent the mean ± Sd of 3 biological replicates. Statistical analysis in **A)** and **B)** was performed using a 2-tailed Student's unpaired *t*-test using Col-0 or *pdx3-3* as control (^ns^*P* > 0.05, *P ≤ 0.005, and ***P ≤ 0.005). Statistical analysis in **C)** and **E)** was performed using a 2-way ANOVA with Tukey's multiple comparisons test (different letters indicate *P* ≤ 0.05). Statistical analysis in **D)** was performed using 1-way ANOVA with Tukey's multiple comparisons test (different letters indicate *P* ≤ 0.05). The analyses were performed on rosettes of plants grown on soil (unfertilized) under a 16-h photoperiod (120 to 190 *µ*mol photons m^−2^ s^−1^) at 22 °C and 8-h darkness at 18 °C. Abbreviation: FC, fold change.

We next examined for a possible connection between SA and vitamin B_6_ homeostasis applicable to the loss of PDX3. We determined the vitamin B_6_ profile of *sid2-1* and *NahG* lines compared to wild type. We noted that there was a significant decrease in PMP, PM, and PL that drove an overall decrease in vitamin B_6_ levels in *NahG* plants in particular compared to wild type (Fig. 8A). Noteworthy, this was also sometimes observed in *sid2-1* plants but was inconsistent. On the other hand, the vitamin B_6_ profile of *pdx3-3 sid2-1* and *pdx3-3 NahG* mutant lines was largely similar to that of the *pdx3-3* single mutant (Fig. 8B). To test if SA may contribute to modulation of vitamin B_6_ contents, we treated wildtype plants with SA and determined the vitamin B_6_ profile. We observed an increase in PM in particular, as well as PL, albeit more modest (Fig. 8C). The increase was transient and had reverted back to the original levels 48 h after treatment, coincident with a visible recovery of the plants from the treatment and repression of the induction of *PR1* (Fig. 8D). To test the importance of the transient response in wild type, we also determined the vitamin B_6_ profile of *Arabidopsis* lines that constitutively accumulate SA and, in this context, phenocopying *pdx3*. In particular, we used the *BONZAI1* mutant (*bon1-1*) and the gain-of-function *SUPRESSOR OF npr1-1*, *CONSTITUTIVE 1* mutant (*snc1-1*) (Hua et al. 2001; Li et al. 2001; Zhang et al. 2003; Yang and Hua 2004). BON1 is a regulator of growth and defense homeostasis that mediates its response by negatively regulating the haplotype-specific *R* gene, *SNC1*. The *bon1-1* line had significantly increased levels of PLP (as well as PM and PL but not always consistent) that drove a modest increase in total vitamin B_6_ levels (Fig. 8E). Interestingly, *snc1-1* plants had significantly increased levels of PMP, PLP, and PL that resulted in an increase in total vitamin B_6_ levels (Fig. 8E).

Taken together, we infer that SA alters vitamin B_6_ metabolism. These effects do not mimic those of *pdx3* plants but may (partially) compensate the vitamin B_6_ imbalance, and thus *pdx3* plants fare better with SA signaling-dependent responses. Nonetheless, *Arabidopsis* plants that lack PDX3 cannot completely overcome the vitamin B_6_ imbalance (increased PMP:PLP) that drives a C/N imbalance and as a consequence overaccumulation of SA at 22 °C on soil, when nitrate is the predominant source of N. Ammonium fertilization or elevated temperatures (at ambient CO_2_) that drive a different metabolism (coincident with reducing SA levels) bypass the requirement for the biochemical function of PDX3.

## Discussion

Here, we build on previous work (Colinas et al. 2016) to highlight the importance of the enzyme PDX3 to metabolic homeostasis under distinct environmental conditions. Under standard ambient conditions for *Arabidopsis*, the absence of PDX3 compromises C/N balance through accumulation of nitrogenous compounds, and SA-related defenses are triggered. Although PDX3 is a 2-domain protein related to NAD(P)H homeostasis and vitamin B_6_ homeostasis, respectively, our study demonstrates that the domain related to vitamin B_6_ homeostasis (POX domain) appears to be the most relevant in terms of C/N balance as dissected in this study. The metabolic imbalance and its negative impact on growth of *pdx3* lines can be fully alleviated by ammonium fertilization or growth of plants under higher temperatures, which as we show here do not appear to rely on the biochemical function of PDX3 due to a different metabolic homeostasis under these conditions. These conditions also dampen the overstimulated SA-related defense response in *pdx3* lines and can be partially mimicked by crossing lines diminished in SA content with *pdx3* lines. However, repression of SA signaling further compromises *pdx3* plant growth, suggesting *pdx3* plants co-opt SA for increased fitness. Intriguingly, our data also show that PDX3 function is particularly important under elevated CO_2_ conditions—a feature associated with serine biosynthesis, thus denoting a distinct function for this protein in its contribution to vitamin B_6_ and C/N balance.

**Figure 9.**
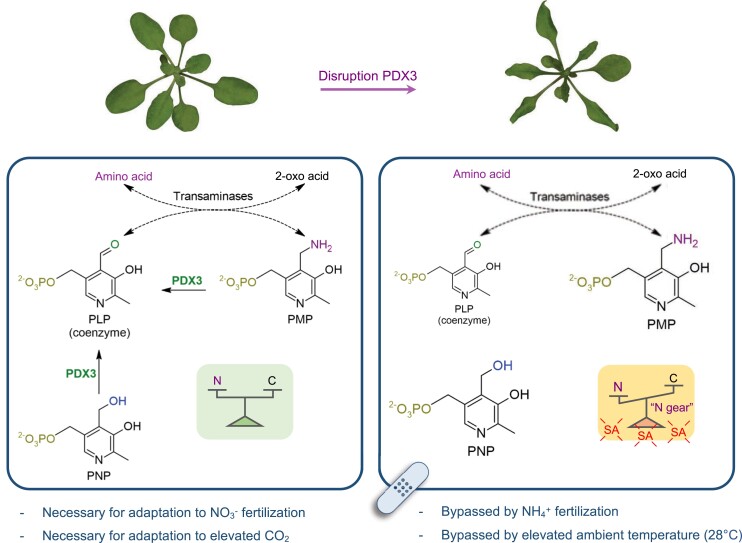
Impact of PDX3 on C/N balance in *Arabidopsis*. Left panel: PDX3 serves to balance the B_6_ vitamers PMP and PLP both of which are coenzyme intermediates during amino acid and 2-oxo acid interconversions by transaminases. This in turn contributes to C and N balance optimal for plant growth, particularly under nitrate $\left({\mathrm{NO}}_{3}^{-}\right)$ fertilization and elevated CO_2_ conditions. PDX3 can also use PNP to form PLP. Right panel: in the absence of PDX3, PMP and PNP accumulate, and there is less PLP that perturbs the C/N equilibrium in favor of N (“N-gear”). This is accompanied by a SA defense response and negatively impacts plant growth and fitness. The morphological phenotype resulting from a lack of PDX3 function can be bypassed (band-aid) by ammonium $\left({\mathrm{NH}}_{4}^{+}\right)$ fertilization or elevated ambient temperatures (28 °C).

We first examined the consequences of N fertilization on the metabolite profile of *pdx3* lines. The suppression of the morphological phenotype of *pdx3* by supplementation with ammonium could be explained as bypassing a nutritional deficiency in *pdx3* lines when nitrate is the source of N. However, this simple explanation is not sufficient to describe observations in this study and suggests a more complex role for PDX3 in managing endogenous N metabolism. Firstly, the metabolite profile of *pdx3* leaves is largely indistinguishable from that of wildtype leaves upon ammonium fertilization, which suggests that metabolism of exogenous ammonium is intact in *pdx3* lines. Assimilation of exogenous ammonium is diagnosed by accumulation of several nitrogenous compounds, e.g. amino acids, reduction of NR activity, as well as an increase in the level of certain B_6_ vitamers such as both PMP and PLP. However, some of these features are diagnostic of *pdx3* in the absence of fertilization. In *pdx3* mutants, PMP increases, but in contrast to wild type, there is a deficit in PLP, and thus the PMP:PLP ratio is not maintained (see scheme in Fig. 9). Notably, inactivation of either the NNRE domain alone involved in (NAD(P)H repair) or the POX domain alone that oxidizes PMP to PLP in PDX3 shows that the POX domain is principally involved in plant homeostasis and if inactivated leads to morphological and metabolic defects. By contrast, inactivation of the NNRE domain alone does not impact the plant. We therefore infer that PDX3 in its role of maintaining vitamin B_6_ homeostasis is important for *Arabidopsis*. During transaminase reactions that allow the interconversion of amino and 2-oxo acids (Koper et al. 2022), PMP is a natural intermediate derived from the coenzyme PLP that facilitates the transfer of an amino group to a 2-oxo acid to make an amino acid (Fig. 9). An increase in PMP levels (as seen in *pdx3* lines) may drive the equilibrium in favor of amino acid formation (which we refer to as the “N-gear,” Fig. 9). Indeed, the observed decreased levels of free ammonium (Fig. 4G) support the notion of increased N assimilation in *pdx3* lines, albeit from endogenous sources (see below). Therefore, we propose that *pdx3* plants suffer a C/N imbalance (Fig. 9), which is supported by the induction of marker genes *ASN1* and *ATL31*. As C skeletons are required also for biosynthesis of nitrogenous compounds, a shift to the “N-gear” in *pdx3* lines would compromise C oxidation for energy generation and would account for the impaired growth of the plants, an association that can be investigated in future studies. Notably, C limitation may also trigger a higher activity of SNF1-related kinase1 (SnRK1) (Mair et al. 2015), which in *pdx3* lines could also explain the observed decreases in protein levels of NR, as its phosphorylation by SnRK1 allows for the anchoring of 14.3.3 proteins that in turn are targets for ubiquitination by ubiquitin ligases such as ATL31 (Lillo et al. 2004; Polge et al. 2008; Huarancca Reyes et al. 2018). Notwithstanding, amino acids are reported to negatively regulate biosynthesis of NR (Oaks et al. 1977). Nitrate levels are similar among wild-type and *pdx3* plants, suggesting that nitrate uptake is not impacted in *pdx3* mutants. The notion that PDX3 is not required for exogenous ammonium assimilation is supported by the recent implication of other vitamin B_6_ genes in this process. Specifically, the biosynthesis de novo gene *PDX1.1* is transcriptionally induced in *Arabidopsis* seedlings grown on medium supplemented with ammonium (Liu Y et al. 2022) and ascribed to the associated management of reactive oxygen species (Swift et al. 2020; Liu Y et al. 2022). Further, the kinase that phosphorylates B_6_ vitamers, SOS4, has been implicated in management of nitric oxide levels that also accompanies exogenous ammonium uptake (Zhang et al. 2021). Such mechanisms under ammonium nutrition would mask the negative effects seen when *PDX3* is absent under nitrate nutrition or on unfertilized soil (where nitrate is the predominant source of N) and thus management of exogenous ammonium likely operates independently of PDX3.

As nitrate assimilation is impaired in *pdx3* lines, we infer that N is sourced internally to furnish accumulation of nitrogenous compounds in *pdx3* lines when grown on soil. N as ammonium can be released and recycled in the plant through photorespiration and the degradation of N compounds such as arginine (Coruzzi 2003; Hildebrandt et al. 2015). Indeed, the accumulation of urea and ornithine in *pdx3* lines indicates arginine degradation, even though steady-state arginine levels are high. Of note also is an early study reporting inhibition of GDH by PLP and inversely promotion of activity in the direction of ammonium release and assimilation into amino acids when PLP levels are low (Teixeira and Davies 1974). The lower levels of PLP in *pdx3* lines could account for the corresponding increase in GDH deaminating activity observed here and contribute to the increase in amino acids. Ammonium release from photorespiration could also fuel amino acid biosynthesis, but the exaggeration of the *pdx3* phenotype under high CO_2_ when photorespiration is diminished (Benstein et al. 2014) allows us to deduce that impaired photorespiration is not the primary cause of the growth phenotype. Although, as nitrate assimilation is reduced under high CO_2_ (Bloom et al. 2010, 2020; Bloom 2015), this could account for the more drastic phenotype under this condition. This is in contrast to the recently described *er-ant1* mutant which suffers a PLP deficiency that impacts photorespiration and is alleviated by growth under high CO_2_ (Altensell et al. 2022). Thus, the *pdx3* phenotype is a consequence of a different metabolic defect. These observations led us to serine biosynthesis and the importance of this amino acid for growth and N metabolism as recently illustrated by the Krueger group (Wulfert and Krueger 2018; Zimmermann et al. 2021). Interestingly, impairment of the PPSB pathway triggers an alteration in N metabolism, exemplified by enhanced ammonium assimilation and an increase in amino acids, as also observed here for *pdx3* lines (Zimmermann et al. 2021). The contribution of PDX3 to this process is supported by the rescue of *pdx3* growth upon exogenous serine supply, particularly under high CO_2_ conditions, when serine biosynthesis through photorespiration in *Arabidopsis* is compromised and the PPSB route that relies on PLP is crucial. Notably, PLP is also a key coenzyme for glycine decarboxylase (P-protein), and moreover, PNP has been reported to inhibit its functionality in bacteria (Ito et al. 2019). The deficit in PLP and increase in PNP observed in *pdx3* lines may thus also limit serine supply through photorespiration which is exasperated under high CO_2_.

Compromising PDX3 biochemical function renders the plant with an autoimmune phenotype as reported previously (Colinas et al. 2016) that reduces growth under the standard temperature of 22 °C. Previous studies on SMALL UBIQUITIN-RELATED MODIFIER (SUMO), *siz1-2* and *siz1-3*, and *nudt6-2 nudt7* have shown that a low ammonium to nitrate ratio triggers SA accumulation and induction of marker transcripts, such as *PR1* that can be alleviated by ammonium fertilization (Park et al. 2011; Wang et al. 2013; Kim et al. 2021). A similar mechanism may operate in *pdx3* lines that compromises growth, as the ammonium content is decreased and nitrate content is similar to that in the wild type under standard growth conditions (22 °C) without fertilization. On the other hand, the wild-type behavior of *pdx3* lines at 28 °C can be explained by triggering of the thermomorphogenesis program mediated by PIF4, which alongside SIZ1-dependent SUMOylation facilitates the downregulation of SNC1-dependent immunity and SA biosynthesis, to favor growth (Gangappa et al. 2017; Hammoudi et al. 2018). In accordance with this, defense gene transcripts and SA were reduced in *pdx3* lines at 28 °C. Initially, we then inferred that the morphological phenotype of *pdx3* is purely a consequence of SA-triggered autoimmunity that may derive from the lower ammonium to nitrate ratio. However, although genetic manipulation to diminish SA levels improved *pdx3* plant growth, it did not completely rescue growth and lesions became visible particularly when the *NahG* transgene was present. This is in contrast to full rescue upon ammonium fertilization or at elevated ambient temperature. To our surprise, *pdx3* lines were even more compromised when the SA receptor NPR1 was genetically removed, i.e. in the *pdx3 npr1* double mutant. Therefore, the signaling response, even from a basal level of SA, is beneficial to *pdx3* lines that suffer a C/N imbalance, assisting growth at 22 °C. Although we demonstrate that SA can alter the vitamin B_6_ profile, the changes (predominantly PM) do not overlap with those of loss of function *pdx3* mutants (increased PMP and decreased PLP). Thus, the increase in SA in *pdx3* lines may be a protective strategy that is consequential to the PMP:PLP imbalance leading to increased N assimilation that lowers the ammonium to nitrate content. Importantly at 28 °C, we saw a shift in metabolism that revealed a higher proportion of C compounds even in wild type, which in *pdx3* lines may balance the C/N imbalance at 22 °C. This observation would be in accordance with the recent report that PIF4-mediated thermomorphogenesis is dependent on sufficient sugar supply, reflected by a higher C status and signaled by trehalose-6-phosphate (T6P) inhibition of the KIN10 kinase subunit of SnRK1 (Hwang et al. 2019). The latter would otherwise (low sugar) phosphorylate PIF4 leading to its degradation (Hwang et al. 2019). We conclude that the thermomorphogenesis program is intact in *pdx3* lines.

## Conclusion

Overall, in the absence of PDX3, our data suggest that metabolic remodeling occurs as a function of the alteration of vitamin B_6_ homeostasis and plants are triggered into the N-gear scavenging endogenous ammonium that compromises NR levels, an effect that is exaggerated by a deficit in PLP-dependent serine biosynthesis. The defects are bypassed when plants are fed with exogenous ammonium or are grown under increased temperature (Fig. 8) due to a different metabolic equilibrium under these conditions that is not reliant on PDX3. Thus, although our study is in *Arabidopsis*, it is possible that PDX3 could undergo negative selection in breeding crops that are overly dependent on ammonium-enriched fertilizers, such as during the green revolution. However, modern and future approaches that strive for the use of less (ammonium-based) fertilizers will likely reveal its indispensability and requirement for N management, particularly when nitrate is a source of N. Moreover, plants that depend on ammonium fertilization are likely compromised in the SA-mediated responses, as SA biosynthesis is reduced under this condition. Furthermore, PDX3 may be an important contributor in breeding for future climates with increased CO_2_ without having to increase ammonium fertilization. Our study suggests that PDX3 provides a surveillance mechanism for PMP:PLP ratios implying that these vitamers are sensed. It will be exciting in the future to unravel how this occurs.

## Materials and methods

### Plant material and growth conditions

*Arabidopsis* (*A. thaliana* Columbia ecotype), *PDX3* (At5g49970) mutants *pdx3-3* (SALK_054167C), and *pdx3-4* (GK-260E03), complementing lines *pdx3-3 35S::PDX3_1* and *pdx3-4 35S::PDX3_1*, were described in Colinas et al. (2016); independent complementing lines *pdx3-3 35S::PDX3_2* and *pdx3-4 35S::PDX3_2* were also included here. The autoimmune lines *bon1-1* described in Hua et al. (2001), *snc1-1* described in Li et al. (2001) were donated by Prof. Jian Hua (Cornell University). The plant line expressing the *NahG* transgene described in Lawton et al. (1995) was donated by Dr. Christiane Nawrath and Prof. Philippe Reymond (University of Lausanne). The *sid2-1* line described in Nawrath and Metraux (1999) was donated by Prof. Roman Ulm (University of Geneva). The *npr1-2* line described in Cao et al. (1997) was obtained from the European Arabidopsis Stock Center (N3801, NASC). For metabolic profiling, GDH activity assay, NR activity assay, nitrate measurement, gene expression, and protein level analysis, plants were grown on soil (Einheitserde, Patzer classic ton Kokos) with a composition of 25% clay, 45% wheat peat, 15% brown peat, and 15% coconut fiber pH 5.8. Before use, the soil was sterilized for 1 h at 60 °C and supplemented once with a 2 ml L^−1^ solution of entomopathogenic nematodes (Andermatt, Traunem) and 2 tablespoons L^−1^ of *Bacillus thuringiensis israelensis* (Andermatt, Solbac). Plants were either watered with tap water (Geneva, Switzerland; −) or supplemented every 9 to 10 d with either a 50 mM solution of potassium chloride (+KCl), potassium nitrate (+KNO_3_) or ammonium nitrate (+NH_4_NO_3_) in equal volumes and grown under a 16-h photoperiod (long-day) at 120 to 190 *µ*mol photons m^−2^ s^−1^ generated by fluorescent lamps at 22 °C and 8-h darkness at 18 °C or constant temperatures of 28 °C with 60% relative humidity and ambient CO_2_ up to 21 DAG. Rosette leaves were harvested 3 h after the onset of illumination for RT-qPCR, metabolite profiling, and GDH activity assay, 3 to 6 h after the onset of illumination for nitrate content and HPLC, and 7 h after the onset of illumination for immunochemical analyses. For phenotypic comparison of plants grown under elevated CO_2_ (HC, 3,000 ppm) or ambient CO_2_ (LC, 380 ppm), a 12-h photoperiod (120 *µ*mol photons m^−2^ s^−1^) at 22 °C combined with 12 h of darkness at 18 °C was used. For free ammonium measurement and serine supplementation, seeds were sown on plates containing modified MS medium containing no ammonium (Sigma-Aldrich M2909), 0.05% (*w*/*v*) MES, 0.01% (*w*/*v*) myoinositol and 0.55% (*w*/*v*) agar. L-serine (100 *µ*M) was added from a 0.1 M filter-sterilized stock solution.

### Generation of transgenic PDX3 active site mutants

Full-length PDX3 including the 5′ and 3′ UTR was amplified from cDNA of *Arabidopsis* plants and cloned into the pENTR/D-TOPO vector using primers shown in Supplemental Table S1 and following the procedures described in (Colinas et al. 2016). Aspartate-238 was substituted to alanine (D238A) within the NNRE domain of PDX3, and arginine-505 was substituted to alanine (R505A) within the POX domain of PDX3 by site-directed mutagenesis on pENTR_PDX3 (see Supplemental Table S1 for primers used) and PfuTurbo DNA Polymerase AD (Agilent). Subsequently, the mutated PDX3 inserts were cloned into the Gateway destination vector pB7YWG2 (Karimi et al. 2002) using LR clonase enzyme mix II (Life Technologies), followed by *Agrobacterium tumefaciens* strain C58-mediated transformation into either *pdx3-3* or *pdx3-4 Arabidopsis* mutant plants by the floral dip method (Clough and Bent 1998). As the respective constructs contain the *BAR* gene, transformants were selected by resistance to BASTA. Resistant plants were allowed to self-fertilize, and homozygous lines were selected from the T3 generation according to their segregation ratio for BASTA resistance. The resulting transgenic lines were additionally verified for the presence of the *PDX3* transgene by PCR analysis of genomic DNA using the same primers as for its amplification from cDNA (Supplemental Table S1) followed by Sanger sequencing (Microsynth AG) of the PCR fragment to verify the respective mutations. Plant lines generated were *pdx3-3 35S::PDX3_D238A*, *pdx3-4 35S::PDX3_D238A*, *pdx3-3 35S::PDX3_*R505A, and *pdx3-4 35S::PDX3_R505A*

### Recombinant expression and purification of PDX3 wild type and mutant

For expression and purification of wild-type PDX3 with an N-terminal hexahistidine tag, we used a construct generated by (Colinas et al. 2014) consisting of *PDX3* (At5g49970) cDNA without the predicted transit peptide (Residues 2 and 73) cloned into the pET-28a vector (Novagen). The mutation consisting of aspartate-238 to alanine (D238A) substitutions within the NNRE domain of PDX3 (numbering based on protein length including the transit peptide) was introduced by site-directed mutagenesis of pET-28a_PDX3 (see Supplemental Table S1 for primers used) and PfuTurbo DNA Polymerase AD (Agilent). For the expression of the dehydratase, NNRD (At5g12150), with a C-terminal hexahistidine tag and no transit peptide (residues 2 to 45), we used a construct generated by (Colinas et al. 2014) consisting of *NNRD* cDNA cloned into a pET-24b vector (Novagen). All constructs were transformed separately into the *Escherichia coli* BL21 (DE3) strain and cultured on LB broth medium supplemented with 50 *µ*g ml^−1^ kanamycin at 37 °C. When the bacterial culture reached an optical density of 0.5 at 600 nm, protein expression was induced by adding 100 *µ*M of isopropyl β-D-1-thiogalactopyranoside followed by incubation with shaking for 3 h at 37 °C. The bacterial pellets were harvested by centrifugation and resuspended in lysis buffer (25 mM Tris–HCl pH 8.0, containing 300 mM sodium chloride, 10 mM imidazole, and 0.1 mM PMSF and protease inhibitor cocktail [Roche]) and lysed by adding lysozyme followed by sonication. The soluble protein was first purified by affinity chromatography with Protino nickel nitrilotriacetic acid (Ni-NTA) (Macherey-Nagel) using the lysis buffer but with the imidazole concentration changed to sequential rounds of 20 mM and 250 mM imidazole for washing and eluting the protein, respectively. Additionally, the proteins were subjected to size exclusion chromatography on a Superdex 200 10/300 increase column (GE Healthcare) and eluted using 25 mM Tris–HCl pH 8.0 containing 100 mM KCl. The purified proteins were used directly for enzymatic assays without further concentrating or freezing/thawing steps.

### Production of NADHX and PDX3 epimerase and NNRD dehydratase assays

As described by (Acheson et al. 1988), NADHX was obtained by dissolving 10 mg of NADH in 500 *µ*l of 0.5 M sodium phosphate buffer pH 6.0 and incubating at 35 °C for 30 min. The reaction was then neutralized by addition of 1 M NaOH until a pH of 8 was reached. The NNRD spectrophotometric assay was carried out as described in (Colinas et al. 2014). Briefly, 0.4 *µ*l of the generated NADHX mixture was added to 25 mM Tris–HCl pH 8, containing 1 mM ATP, 0.1 mg ml^−1^ BSA, 2 mM MgCl_2_, and 5 mM KCl to reach a total volume of 100 *µ*l when enzyme solutions were included. The mixture was allowed to equilibrate at 25 °C for 30 min, then 0.125 *µ*M NNRD (or buffer as a no enzyme control) was added, and the reaction followed at 290 nm (NADHX consumption) and 340 nm (NADH production) for 1 h at 25 °C in a plate reader (BioTek Synergy2). Once a new stable line was reached, 0.125 *µ*M of either wild-type PDX3 or PDX3 D238A (or buffer as a no enzyme control) was added in separate reactions and the consumption of NADHX and NADH measured as described above for 1 h.

### Generation of double mutants

The *pdx3-3 NahG* and *pdx3-3 sid2-1* double mutants were generated using pollen from a *pdx3-3* plant to fertilize flowers of *NahG* and *sid2-1* plants. The *pdx3-4 NahG* and *pdx3-4 sid2-1* double mutants were generated in the same way by using the pollen of a *pdx3-4* plant. The *npr1-2 pdx3-3* double mutant, on the other hand, was generated using pollen from a *npr1-2* plant to fertilize flowers of a *pdx3-3* plant. The success of the cross was validated by the absence of a *pdx3*-like leaf phenotype of the F1 plants (due to the presence of 1 wild-type copy of *PDX3*). The F2 plants were screened for homozygosity of the respective mutations by analyzing the phenotype frequency of their F3 offspring and by genotyping. The presence of the T-DNA insertion in *pdx3-3*, *pdx3-4*, or the *NahG* transgene was verified by PCR analysis of genomic DNA using oligonucleotides reported in Supplemental Table S1. The presence of a point mutation in *sid2-1* and *npr1-2* was verified by PCR analysis of genomic DNA using oligonucleotides reported in Supplemental Table S1, followed by Sanger sequencing of the PCR fragment (Microsynth AG).

### Immunochemical analyses

Five volumes of extraction buffer (50 mM Tris–HCl pH 8.5, containing 10 mM EDTA, 0.1% Triton X-100 [*v*/*v*], 10% glycerol [*v*/*v*], and 1% [*v*/*v*] complete plant protease inhibitor cocktail [P9599, Sigma-Aldrich]) were added to frozen and ground plant material. After brief homogenization, samples were centrifuged for 15 min at 10,000 × *g* at 4 °C, and the supernatant was collected. The protein concentration was determined by the Bradford method (Bradford 1976). Samples were then separated by 10% SDS-PAGE loading 5 to 25 *µ*g of total protein per lane. For western blot analysis, the proteins were transferred onto nitrocellulose membranes using the iBlot system (Invitrogen) for a total of 8 min applying 20 V for 1 min followed by 23 V for 4 min and finally 25 V for the remainder of the time. The membranes were stained with Ponceau S (0.1% Ponceau S [*w*/*v*] in 5% acetic acid [*v*/*v*]) to confirm protein transfer before removing the stain by washing in Tris buffered saline +0.1% Tween (*v*/*v*) (TBS-T). The following primary antibodies and dilutions were used: α-PDX3 as described in (Colinas et al. 2014) 1:3,000; α-NR (AS08310, Agrisera); 1:10,000; α-ACTIN-2 as loading control (A0480, Sigma-Aldrich); and 1:10,000. The secondary antibody Goat Anti-Rabbit IgG (H + L)-HRP (1706515, Bio-Rad) was used in combination with α-PDX3 at a dilution of 1:3,000 and α-NR at a dilution of 1:10,000. For α-ACTIN-2, the secondary antibody Goat Anti-Mouse IgG (H + L)-HRP conjugate (1706516, Bio-Rad) was used at a dilution of 1:5,000. The immunoblot analysis of PDX3 and ACTIN-2 was performed using a SNAP i.d. 2.0 system (Millipore) as described in (Colinas et al. 2014). For NR analysis, blots were blocked in 5% (*w*/*v*) nonfat milk dissolved in TBS-T for 1 h at 22 °C with agitation followed by incubation in the primary antibody (in blocking reagent) for 1 h at 22 °C with agitation. The antibody solution was removed, and the blot was washed 1 × 15 min and 3 × 5 min with TBS-T at 22 °C with agitation. Thereafter, blots were incubated in secondary antibody (in blocking reagent) for 1 h at 22 °C with agitation and washed as described above. Chemiluminescence was detected using ECL SuperBright (AS16 ECL-S, Agrisera) for NR and Western Bright ECL (K-12045, Advansta) for PDX3 and ACTIN-2 and captured using an Amersham Imager 680 system (GE Healthcare).

### Nitrate and free ammonium measurements

Nitrate determination was based on (Zhao and Wang 2017). Briefly, for extraction, 10 vol of deionized water was added to frozen and ground plant material, and samples were homogenized and heated at 100 °C for 30 min. The samples were centrifuged for 10 min at 15,000 × *g*, and the supernatants were decanted. A standard curve of 10 to 120 mg L^−1^ KNO_3_ was used. For the reaction, 10-*µ*l sample or standard was mixed with 40 *µ*l of 5% (*w*/*v*) salicylic acid (in H_2_SO_4_) and incubated at room temperature for 30 min. Thereafter, a yellow-color complex was revealed after addition of 950 *µ*l of 8% (*w*/*v*) of NaOH, 200 *µ*l of the mixture was transferred to a 96-well plate, and the OD_410_ was measured using a Synergy2 microplate reader (BioTek). Free ammonium in leaves was determined by the indophenol-blue reaction as described in (Scheiner 1976) with adaptations. For extraction, 1 ml of 100 mM HCl was added to 100-mg frozen and ground plant material, samples were homogenized, and 500 *µ*l of chloroform was added. The samples were shaken for 15 min at 4 °C and centrifuged for 10 min at 12,000 × *g* at 8 °C. The supernatant was decanted and transferred to a 1.5-ml Eppendorf tube containing 50 mg of acid-washed activated charcoal thoroughly vortexed and centrifuged for 5 min at 20,000 × *g* at 8 °C. The supernatant was decanted once more, transferred to a fresh tube and centrifuged again for 10 min at 20,000 × *g* at 8 °C and the supernatant decanted again. A standard curve of 0 to 100 *µ*M (NH_4_)_2_SO_4_ was used. For the reaction, equal volumes of sample or standard and 100 mM HCl were mixed. In a 96-well plate, 20 *µ*l of the sample mixture was added to 100 *µ*l of Solution I (1% (*w*/*v*) phenol and 0.005% (*w*/*v*) sodium nitroprusside) followed by the addition of 100 *µ*l of Solution II (1% (*w*/*v*) sodium hypochlorite, 0.5% (*w*/*v*) sodium hydroxide). The plate was sealed with Parafilm and incubated at 37 °C for 30 min, and OD_620_ was measured using a Synergy H1 microplate reader (BioTek).

### Gene expression analysis by RT-qPCR

RNA was extracted using the RNA NucleoSpin Plant kit (Macherey-Nagel) according to the manufacturers’ instructions. Reverse transcription (RT) was performed using 0.5 to 1 *µ*g of total RNA as template and Superscript II (Invitrogen) according to the instructions with the following modifications: stock oligo(dT)_15_ primer (C1101, Promega) concentration was 50 ng/*µ*l, and 0.5-*µ*l Superscript II enzyme was used per reaction. RT-qPCR was performed in 384-well plates on an Applied Biosystems QuantStudio 5 qPCR-System (Thermo Fisher Scientific) using PowerUp SYBR Green master mix (A25743, Applied Biosystems) and the following amplification program: 10-min denaturation at 95 °C followed by 40 cycles of 95 °C for 15 s and 60 °C for 1 min. The data from nitrogen supplementation experiments were analyzed using the comparative cycle threshold method (2^−ΔCT^ or 2^−ΔΔCT^) normalized to the reference gene *ACT2* (At3g18780) and *UBC21* (At5g25760), whereas only *UBC21* was used in other experiments. Oligonucleotide pairs used are indicated in Supplemental Table S1. The 2 primer pairs for *GDH2* and *ATL31* gave similar results in independent experiments using the same data set.

### GDH activity

The animating (NADH-dependent) activity of GDH was determined as described in (Turano et al. 1996) except that the extraction buffer consisted of 100 mM Tris–HCl pH 7.6, containing 1 mM MgCl_2_, 1 mM EDTA, and 14 mM β-mercaptoethanol.

### Vitamin B_6_ analysis by HPLC

Vitamin B_6_ profiling was performed as described in (Colinas et al. 2016) with the following changes: 2 separate extractions were performed with 15 volumes and 2 to 3 volumes of 50 mM ammonium acetate (pH 4), respectively, and a 50-*µ*l injection volume was used for a single run per extract.

### Gas exchange measurements

Col-0 and *pdx3* mutants were grown under 12-h light (100 to 150 *µ*mol photons m^2^ s^−1^) at 22 °C and 12-h dark cycles at 18 °C to promote vegetative growth at ambient air. For measuring the oxygen sensitivity of photosynthesis, oxygen concentrations at 2%, 20%, and 40% were generated as oxygen/nitrogen mixes by a gas-mixing system (Vögtlin Instruments). The plants were acclimated for 20 min, and the net photosynthetic rate was measured at 400 *µ*l L^−1^ CO_2_ using an LI-6400XT (LiCor) photosynthesis analyzer.

### Metabolite profiling

For each analysis, 6 experimental replicates of Col-0, *pdx3-3*, and *pdx3-4* were grown under long-day conditions. For the analysis of N supplementation, plants were either watered normally or supplemented every 9 to 10 d with a 50 mM solution of either KNO_3_ or NH_4_NO_3_ as indicated and harvested when they were 21 d old. The material of 4 to 6 plants was pooled and ground in liquid nitrogen using a mortar and a pestle. For data on the effect of high temperature, plants were subjected to a constant temperature of 28 °C or to a temperature of 22 °C during the photoperiod and 18 °C during darkness and harvested when they were 12 d old and 14 d old, respectively. For the analysis of *pdx3-3* and its complementing lines (*pdx3-3 35S::PDX3_D238A* and *pdx3-3 35S::PDX3_*R505A), plants were grown under long-day conditions with no additional fertilization and harvested when they were 14 d old. The material of 12 plants was pooled and ground in liquid nitrogen using a mortar and a pestle. The resulting powder was weighed and stored at −80 °C to be used for GC-MS as described in (Lisec et al. 2006) with peak annotation based on libraries of authentic standards (Kopka et al. 2005).

### SA treatment

Rosette leaves of 19- to 20-d-old soil-grown Col-0 plants were sprayed equally with a solution of 0.005% (*v*/*v*) Silwet L77 only as a mock treatment or containing 2 mM SA (247588, Sigma-Aldrich). Treatment was started 0.5 h after the onset of light. Whole rosette leaves were harvested in liquid nitrogen in triplicate (pools of 5 to 8 plants) in a time series of 3, 12, 24, and 48 h after treatment. All samples were harvested during the photoperiod.

### NR activity

For NR activity measurements with the purified protein, the procedure was adapted from Sigma quality control tests for product N7265 based on Gilliam et al. (1993). *Arabidopsis* NR2 (NIA2, At1g37130, ≥ 0.5 units of NR per vial) expressed and purified from *Pichia pastoris* was purchased from Sigma-Aldrich (N0163). The lyophilized protein was resuspended in 50 *µ*l of 57 mM potassium phosphate at pH 7.5 and was used directly or stored at −80 °C. Before the start of the assay, the NR enzyme was diluted 100× in 57 mM potassium phosphate at pH 7.5 (≥0.01 units/*µ*l). To circumvent the overlap in absorbance between PMP (82890, Sigma-Aldrich) and the NADH coenzyme, the latter was replaced with its analog, 3-acetylpyridine-adenine dinucleotide (APADH, MBS682175 MyBiosource). The reaction was carried out in a 96-well plate in 57 mM potassium phosphate pH 7.5 containing 0.005 mM FAD, 10 mM potassium nitrate, 0.1 mM APADH, and 0 to 1 mM PMP and was started by the addition of 50 *µ*l of NR (≥ 0.005 units added) for a total reaction volume of 200 *µ*l. Alternatively, the reaction mixture without potassium nitrate was incubated for 25 min at 25 °C before starting the reaction with the addition of 20 *µ*l of 100 mM potassium nitrate (10 mM final concentration). The reaction was followed by measuring the absorbance at 363 nm at 25 °C for 15 min using a plate reader (BioTek Synergy2). For NR activity measurements on material extracted from *Arabidopsis*, the procedure described in Colinas et al. (2016) was used with modifications. Whole rosette leaves of 21-d-old plants grown under long-day conditions were harvested in liquid nitrogen at 0 h (in the dark); 3, 6, and 12 h, after the onset of light; and 2 h after the onset of the following period of darkness. For extraction, 50 *µ*l of extraction buffer (250 mM Tris–HCl, pH 8.0 containing 1 mM EDTA, 5 *µ*M FAD, 1 *µ*M sodium molybdate, 3 mM DTT, 1% [*v*/*v*] Triton-X-100, and 1% [*v*/*v*] plant protease inhibitor cocktail [P9599 Sigma-Aldrich]) was added to 25 mg of frozen and ground plant tissue. After brief homogenization (samples kept on ice), samples were centrifuged 15 min at 13,000 × *g* at 4 °C, and the supernatant was collected. When necessary, to further clear the extract from cell debris, the collected supernatant was centrifuged once more for 5 min at 13,000 × *g* at 4 °C. Using 96-well plates, 10 *µ*l of plant extract or standard was added to 70 *µ*l of 100 mM sodium phosphate pH 7.4 containing 40 mM sodium nitrate, and the reaction was started with the addition of 20 *µ*l of 1 mM NADH (0.2 mM final concentration) for a total reaction volume of 100 *µ*l. The reaction was incubated in the dark at 25 °C and was stopped immediately at 15 to 17, 30, and 45 min after the start of the reaction by adding 50 *µ*l of stop solution (1:1 mixture of 1% [*w*/*v*] sulfanilamide and 0.05% [*w*/*v*] napthylethylenediamine). The absorbance at 540 nm was measured in a Synergy2 microplate reader (BioTek).

### Analysis software

Data rendering and statistical analysis were performed using GraphPad Prism version 8.3.0 for Windows, GraphPad Software, San Diego, California USA, www.graphpad.com. Image rendering was performed using Inkscape 0.92.4 https://www.inkscape.org. Protein quantification was performed using ImageJ https://imagej.nih.gov/ij/. Photo color was modified for homogeneity using https://www.pixelmator.com/pro/.

### Accession numbers

Accession numbers from this article can be found in Supplemental Table S1.

## Supplementary Material

kiad411_Supplementary_Data

## Data Availability

Data is available upon reasonable request from the corresponding author.

## Supplementary data

**Supplemental Fig. S1.** Probing the 2-domain structure of PDX3.

**Supplemental Fig. S2.** Phenotype of rosette leaves of *pdx3* lines compared to wild type.

**Supplemental Fig. S3.** NR activity and *PDX3* expression as a function of PMP and N fertilization, respectively.

**Supplemental Fig. S4.** Photograph of the leaves of wild-type (Col-0) and *pdx3* lines grown up to 14 DAG under the standard temperature of 22 °C compared to 12 DAG under 28 °C.

**Supplemental Table S1.** Oligonucleotides used in this study.

## References

[kiad411-B1] Acheson SA , Kirkman HN, Wolfenden R. Equilibrium of 5,6-hydration of NADH and mechanism of ATP-dependent dehydration. Biochemistry 1988:27(19):7371–7375. 10.1021/bi00419a030 3061454

[kiad411-B2] Altensell J , Wartenberg R, Haferkamp I, Hassler S, Scherer V, Steensma P, Fitzpatrick TB, Sharma A, Sandoval-Ibañez O, Pribil M, et al Loss of a pyridoxal-phosphate phosphatase rescues *Arabidopsis* lacking an endoplasmic reticulum ATP carrier. Plant Physiol. 2022:189(1):49–65. 10.1093/plphys/kiac048 35139220 PMC9070803

[kiad411-B3] Barile A , Nogués I, di Salvo ML, Bunik V, Contestabile R, Tramonti A. Molecular characterization of pyridoxine 5′-phosphate oxidase and its pathogenic forms associated with neonatal epileptic encephalopathy. Sci Rep. 2020:10(1):13621. 10.1038/s41598-020-70598-7 32788630 PMC7424515

[kiad411-B4] Benstein RM , Ludewig K, Wulfert S, Wittek S, Gigolashvili T, Frerigmann H, Gierth M, Flügge UI, Krueger S. *Arabidopsis* phosphoglycerate dehydrogenase1 of the phosphoserine pathway is essential for development and required for ammonium assimilation and tryptophan biosynthesis. Plant Cell 2014:25(12):5011–5029. 10.1105/tpc.113.118992 PMC3904002 24368794

[kiad411-B5] Bloom AJ . Photorespiration and nitrate assimilation: a major intersection between plant carbon and nitrogen. Photosynth Res. 2015:123(2):117–128. 10.1007/s11120-014-0056-y 25366830

[kiad411-B6] Bloom AJ , Burger M, Asensio JS, Cousins AB. Carbon dioxide enrichment inhibits nitrate assimilation in wheat and *Arabidopsis*. Science 2010:328(5980):899–903. 10.1126/science.1186440 20466933

[kiad411-B7] Bloom AJ , Kasemsap P, Rubio-Asensio JS. Rising atmospheric CO_2_ concentration inhibits nitrate assimilation in shoots but enhances it in roots of C_3_ plants. Physiol Plant. 2020:168(4):963–972. 10.1111/ppl.13040 31642522

[kiad411-B8] Bradford MM . A rapid and sensitive method for the quantitation of microgram quantities of protein utilizing the principle of protein-dye binding. Anal Chem. 1976:72:248–254. 10.1016/0003-2697(76)90527-3 942051

[kiad411-B9] Cao H , Glazebrook J, Clarke JD, Volko S, Dong X. The *Arabidopsis NPR1* gene that controls systemic acquired resistance encodes a novel protein containing ankyrin repeats. Cell 1997:88(1):57–63. 10.1016/S0092-8674(00)81858-9 9019406

[kiad411-B10] Chalk P , Smith C. On inorganic N uptake by vascular plants: can ^15^N tracer techniques resolve the NH_4_^+^ versus NO_3_^−^ “preference” conundrum? Eur J Soil Sci. 2021:72(4):1762–1779. 10.1111/ejss.13069

[kiad411-B11] Clough SJ , Bent AF. Floral dip: a simplified method for *Agrobacterium*-mediated transformation of *Arabidopsis thaliana*. Plant J. 1998:16(6):735–743. 10.1046/j.1365-313x.1998.00343.x 10069079

[kiad411-B12] Colinas M , Eisenhut M, Tohge T, Pesquera M, Fernie AR, Weber AP, Fitzpatrick TB. Balancing of B_6_ vitamers is essential for plant development and metabolism in *Arabidopsis*. Plant Cell 2016:28(2):439–453. 10.1105/tpc.15.01033 26858304 PMC4790880

[kiad411-B13] Colinas M , Fitzpatrick TB. Interaction between vitamin B_6_ metabolism, nitrogen metabolism and autoimmunity. Plant Signal Behav. 2016:11(4):e1161876. 10.1080/15592324.2016.1161876 PMC4883958 27018849

[kiad411-B14] Colinas M , Shaw HV, Loubéry S, Kaufmann M, Moulin M, Fitzpatrick TB. A pathway for repair of NAD(P)H in plants. J Biol Chem. 2014:289(21):14692–14706. 10.1074/jbc.M114.556092 24706747 PMC4031525

[kiad411-B15] Coruzzi GM . Primary N-assimilation into amino acids in *Arabidopsis*. In: The Arabidopsis book. Rockville (MD): The American Society of Plant Biologists; 2003. p. 1–17. 10.1199/tab.0010 PMC3243381 22303223

[kiad411-B16] Di Salvo ML , Safo MK, Musayev FN, Bossa F, Schirch V. Structure and mechanism of *Escherichia coli* pyridoxine 5′-phosphate oxidase. Biochim Biophys Acta. 2003:1647(1–2):76–82. 10.1016/S1570-9639(03)00060-8 12686112

[kiad411-B17] Eisenhut M , Roell MS, Weber APM. Mechanistic understanding of photorespiration paves the way to a new green revolution. New Phytol. 2019:223(4):1762–1769. 10.1111/nph.15872 31032928

[kiad411-B18] Gangappa SN , Berriri S, Kumar SV. PIF4 coordinates thermosensory growth and immunity in *Arabidopsis*. Curr Biol. 2017:27(2):243–249. 10.1016/j.cub.2016.11.012 28041792 PMC5266789

[kiad411-B19] Gilliam MB , Sherman MP, Griscavage JM, Ignarro LJ. A spectrophotometric assay for nitrate using NADPH oxidation by *Aspergillus* nitrate reductase. Anal Biochem. 1993:212(2):359–365. 10.1006/abio.1993.1341 8214577

[kiad411-B20] González E , Danehower D, Daub ME. Vitamer levels, stress response, enzyme activity, and gene regulation of *Arabidopsis* lines mutant in the pyridoxine/pyridoxamine 5′-phosphate oxidase (*PDX3*) and the pyridoxal kinase (*SOS4*) genes involved in the vitamin B6 salvage pathway. Plant Physiol. 2007:145(3):985–996. 10.1104/pp.107.105189 17873088 PMC2048783

[kiad411-B21] Gorelova V , Colinas M, Dell’Aglio E, Flis P, Salt DE, Fitzpatrick TB. Phosphorylated B6 vitamer deficiency in SALT OVERLY SENSITIVE 4 mutants compromises shoot and root development. Plant Physiol. 2022:188(1):220–240. 10.1093/plphys/kiab475 34730814 PMC8774746

[kiad411-B22] Hammoudi V , Fokkens L, Beerens B, Vlachakis G, Chatterjee S, Arroyo-Mateos M, Wackers PFK, Jonker MJ, van den Burg HA. The *Arabidopsis* SUMO E3 ligase SIZ1 mediates the temperature dependent trade-off between plant immunity and growth. PLoS Genet. 2018:14(1):e1007157. 10.1371/journal.pgen.1007157 PMC5794169 29357355

[kiad411-B23] He Z , Webster S, He SY. Growth-defense trade-offs in plants. Curr Biol. 2022:32(12):R634–R639. 10.1016/j.cub.2022.04.070 35728544

[kiad411-B24] Herrero S , González E, Gillikin JW, Vélëz H, Daub ME. Identification and characterization of a pyridoxal reductase involved in the vitamin B6 salvage pathway in *Arabidopsis*. Plant Mol Biol. 2011:76(1–2):157–169. 10.1007/s11103-011-9777-x 21533842

[kiad411-B25] Hildebrandt TM , Nunes Nesi A, Araújo WL, Braun HP. Amino acid catabolism in plants. Mol Plant. 2015:8(11):1563–1579. 10.1016/j.molp.2015.09.005 26384576

[kiad411-B26] Hua J , Grisafi P, Cheng SH, Fink GR. Plant growth homeostasis is controlled by the *Arabidopsis BON1* and *BAP1* genes. Genes Dev. 2001:15(17):2263–2272. 10.1101/gad.918101 11544183 PMC312777

[kiad411-B27] Huarancca Reyes T , Scartazza A, Pompeiano A, Ciurli A, Lu Y, Guglielminetti L, Yamaguchi J. Nitrate reductase modulation in response to changes in C/N balance and nitrogen source in *Arabidopsis*. Plant Cell Physiol. 2018:59(6):1248–1254. 10.1093/pcp/pcy065 29860377

[kiad411-B28] Hwang G , Kim S, Cho J, Paik I, Kim J, Oh E. Trehalose-6-phosphate signaling regulates thermoresponsive hypocotyl growth in *Arabidopsis thaliana*. EMBO Rep. 2019:20(10):e47828. 10.15252/embr.201947828 31393060 PMC6776909

[kiad411-B29] Ito T , Yamamoto K, Hori R, Yamauchi A, Downs DM, Hemmi H, Yoshimura T. Conserved pyridoxal 5′-phosphate-binding protein YggS impacts amino acid metabolism through pyridoxine 5′-phosphate in *Escherichia coli*. Appl Environ Microbiol. 2019:85(11):e00430. 10.1128/AEM.00430-19 30902856 PMC6532037

[kiad411-B30] Karimi M , Inzé D, Depicker A. GATEWAY Vectors for Agrobacterium-mediated plant transformation. Trends Plant Sci. 2002:7(5):193–195. 10.1016/S1360-1385(02)02251-3 11992820

[kiad411-B31] Kim JY , Park BS, Park SW, Lee HY, Song JT, Seo HS. Nitrate reductases are relocalized to the nucleus by AtSIZ1 and their levels are negatively regulated by COP1 and ammonium. Int J Mol Sci. 2018:19(4):1202. 10.3390/ijms19041202 29662028 PMC5979280

[kiad411-B32] Kim JY , Song JT, Seo HS. Ammonium-mediated reduction in salicylic acid content and recovery of plant growth in *Arabidopsis siz1* mutants is modulated by NDR1 and NPR1. Plant Signal Behav. 2021:16(9):1928819. 10.1080/15592324.2021.1928819 PMC8281091 33989128

[kiad411-B33] Koper K , Han SW, Pastor DC, Yoshikuni Y, Maeda HA. Evolutionary origin and functional diversification of aminotransferases. J Biol Chem. 2022:298(8):102122. 10.1016/j.jbc.2022.102122 35697072 PMC9309667

[kiad411-B34] Kopka J , Schauer N, Krueger S, Birkemeyer C, Usadel B, Bergmüller E, Dörmann P, Weckwerth W, Gibon Y, Stitt M, et al GMD@CSB.DB: the Golm Metabolome Database. Bioinformatics 2005:21(8):1635–1638. 10.1093/bioinformatics/bti236 15613389

[kiad411-B35] Lam HM , Peng SS, Coruzzi GM. Metabolic regulation of the gene encoding glutamine-dependent asparagine synthetase in *Arabidopsis thaliana*. Plant Physiol. 1994:106(4):1347–1357. 10.1104/pp.106.4.1347 7846154 PMC159672

[kiad411-B36] Lawton K , Weymann K, Friedrich L, Vernooij B, Uknes S, Ryals J. Systemic acquired resistance in *Arabidopsis* requires salicylic acid but not ethylene. Mol Plant-Microbe Interact. 1995:8(6):863–870. 10.1094/MPMI-8-0863 8664495

[kiad411-B37] Li X , Clarke JD, Zhang Y, Dong X. Activation of an EDS1-mediated *R*-gene pathway in the *snc1* mutant leads to constitutive, NPR1-independent pathogen resistance. Mol Plant-Microbe Interact. 2001:14(10):1131–1139. 10.1094/MPMI.2001.14.10.1131 11605952

[kiad411-B38] Lillo C , Meyer C, Lea US, Provan F, Oltedal S. Mechanism and importance of post-translational regulation of nitrate reductase. J Exp Bot. 2004:55(401):1275–1282. 10.1093/jxb/erh132 15107452

[kiad411-B39] Lisec J , Schauer N, Kopka J, Willmitzer L, Fernie AR. Gas chromatography mass spectrometry-based metabolite profiling in plants. Nat Protoc. 2006:1(1):387–396. 10.1038/nprot.2006.59 17406261

[kiad411-B40] Liu Z , Farkas P, Wang K, Kohli MO, Fitzpatrick TB. B vitamin supply in plants and humans: the importance of vitamer homeostasis. Plant J. 2022:111(3):662–682. 10.1111/tpj.15859 35673947 PMC9544542

[kiad411-B41] Liu Y , Maniero RA, Giehl RFH, Melzer M, Steensma P, Krouk G, Fitzpatrick TB, von Wirén N. PDX1.1-dependent biosynthesis of vitamin B6 protects roots from ammonium-induced oxidative stress. Mol Plant. 2022:15(5):820–839. 10.1016/j.molp.2022.01.012 35063660

[kiad411-B42] Mair A , Pedrotti L, Wurzinger B, Anrather D, Simeunovic A, Weiste C, Valerio C, Dietrich K, Kirchler T, Nägele T, et al SnRK1-triggered switch of bZIP63 dimerization mediates the low-energy response in plants. Elife 2015:4:e05828. 10.7554/eLife.05828 26263501 PMC4558565

[kiad411-B43] Marbaix AY , Chehade G, Noël G, Morsomme P, Vertommen D, Bommer GT, Van Schaftingen E. Pyridoxamine-phosphate oxidases and pyridoxamine-phosphate oxidase-related proteins catalyze the oxidation of 6-NAD(P)H to NAD(P)+. Biochem J. 2019:476(20):3033–3052. 10.1042/BCJ20190602 31657440

[kiad411-B44] Nair A , Goyal I, Voß E, Mrozek P, Prajapati S, Thurow C, Tietze L, Tittmann K, Gatz C. N-hydroxypipecolic acid-induced transcription requires the salicylic acid signaling pathway at basal SA levels. Plant Physiol. 2021:187(4):2803–2819. 10.1093/plphys/kiab433 34890459 PMC8644824

[kiad411-B45] Nawrath C , Metraux J-P. Salicylic acid induction-deficient mutants of *Arabidopsis* express *PR-2* and *PR-5* and accumulate high levels of camalexin after pathogen inoculation. Plant Cell 1999:11(1–2):1393–1404. 10.1105/tpc.11.8.1393 10449575 PMC144293

[kiad411-B46] Niehaus TD , Elbadawi-Sidhu M, Huang L, Prunetti L, Gregory F Jr, de Crécy-Lagard V, Fiehn O, Hanson AD. Evidence that the metabolite repair enzyme NAD(P)HX epimerase has a moonlighting function. Biosci Rep. 2018:38(3):BSR20180223. 10.1042/BSR20180223 PMC5938422 29654173

[kiad411-B47] Niehaus TD , Richardson LG, Gidda SK, ElBadawi-Sidhu M, Meissen JK, Mullen RT, Fiehn O, Hanson AD. Plants utilize a highly conserved system for repair of NADH and NADPH hydrates. Plant Physiol. 2014:165(1):52–61. 10.1104/pp.114.236539 24599492 PMC4012604

[kiad411-B48] Nunes-Nesi A , Fernie AR, Stitt M. Metabolic and signaling aspects underpinning the regulation of plant carbon nitrogen interactions. Mol Plant. 2010:3(6):973–996. 10.1093/mp/ssq049 20926550

[kiad411-B49] Oaks A , Aslam M, Boesel I. Ammonium and amino acids as regulators of nitrate reductase in corn roots. Plant Physiol. 1977:59(3):391–394. 10.1104/pp.59.3.391 16659859 PMC542410

[kiad411-B50] Park BS , Song JT, Seo HS. *Arabidopsis* nitrate reductase activity is stimulated by the E3 SUMO ligase AtSIZ1. Nat Commun. 2011:2(1):400. 10.1038/ncomms1408 21772271 PMC3160146

[kiad411-B51] Patterson K , Cakmak T, Cooper A, Lager I, Rasmusson AG, Escobar MA. Distinct signalling pathways and transcriptome response signatures differentiate ammonium- and nitrate-supplied plants. Plant Cell Environ. 2010:33(9):1486–1501. 10.1111/j.1365-3040.2010.02158.x 20444219 PMC2920365

[kiad411-B52] Polge C , Jossier M, Crozet P, Gissot L, Thomas M. *β*-subunits of the SnRK1 complexes share a common ancestral function together with expression and function specificities; physical interaction with nitrate reductase specifically occurs via AKIN*β*1-subunit. Plant Physiol. 2008:148(3):1570–1582. 10.1104/pp.108.123026 18768910 PMC2577271

[kiad411-B53] Poore J , Nemecek T. Reducing food's environmental impacts through producers and consumers. Science 2018:360(6392):987–992. 10.1126/science.aaq0216 29853680

[kiad411-B54] Robinson GC , Kaufmann M, Roux C, Fitzpatrick TB. Structural definition of the lysine swing in *Arabidopsis thaliana* PDX1: intermediate channeling facilitating vitamin B_6_ biosynthesis. Proc Natl Acad Sci U S A. 2016:113(40):E5821–E5829. 10.1073/pnas.1608125113 27647886 PMC5056094

[kiad411-B55] Sang Y , Barbosa JM, Wu H, Locy RD, Singh NK. Identification of a pyridoxine (pyridoxamine) 5′-phosphate oxidase from *Arabidopsis thaliana*. FEBS Lett. 2007:581(3):344–348. 10.1016/j.febslet.2006.12.028 17224143

[kiad411-B56] Sato T , Maekawa S, Yasuda S, Sonoda Y, Katoh E, Ichikawa T, Nakazawa M, Seki M, Shinozaki K, Matsui M, et al CNI1/ATL31, A RING-type ubiquitin ligase that functions in the carbon/nitrogen response for growth phase transition in *Arabidopsis* seedlings. Plant J. 2009:60(5):852–864. 10.1111/j.1365-313X.2009.04006.x 19702666

[kiad411-B57] Scheiner D . Determination of ammonia and Kjeldahl nitrogen by indophenol method. Water Res. 1976:10(1):31–36. 10.1016/0043-1354(76)90154-8

[kiad411-B58] Shi H , Xiong L, Stevenson B, Lu T, Zhu JK. The *Arabidopsis salt overly sensitive 4* mutants uncover a critical role for vitamin B6 in plant salt tolerance. Plant Cell 2002:14(3):575–588. 10.1105/tpc.010417 11910005 PMC150580

[kiad411-B59] Shi H , Zhu JK. *SOS4*, a pyridoxal kinase gene, is required for root hair development in *Arabidopsis*. Plant Physiol. 2002:129(2):585–593. 10.1104/pp.001982 12068103 PMC161684

[kiad411-B60] Shumilin IA , Cymborowski M, Chertihin O, Jha KN, Herr JC, Lesley SA, Joachimiak A, Minor W. Identification of unknown protein function using metabolite cocktail screening. Structure 2012:20(10):1715–1725. 10.1016/j.str.2012.07.016 22940582 PMC3472112

[kiad411-B61] Subbarao GV , Searchinger TD. A “more ammonium solution” to mitigate nitrogen pollution and boost crop yields. Proc Natl Acad Sci U S A 2021:118(22):e2107576118. 10.1073/pnas.2107576118 PMC8179215 34039714

[kiad411-B62] Swift J , Alvarez JM, Araus V, Gutiérrez RA, Coruzzi GM. Nutrient dose-responsive transcriptome changes driven by Michaelis–Menten kinetics underlie plant growth rates. Proc Natl Acad Sci U S A 2020:117(23):12531–12540. 10.1073/pnas.1918619117 32414922 PMC7293603

[kiad411-B63] Szydlowski N , Bürkle L, Pourcel L, Moulin M, Stolz J, Fitzpatrick TB. Recycling of pyridoxine (vitamin B6) by PUP1 in *Arabidopsis*. Plant J. 2013:75(1):40–52. 10.1111/tpj.12195 23551747

[kiad411-B64] Tambasco-Studart M , Tews I, Amrhein N, Fitzpatrick TB. Functional analysis of PDX2 from *Arabidopsis*, a glutaminase involved in vitamin B6 biosynthesis. Plant Physiol. 2007:144(2):915–925. 10.1104/pp.107.096784 17468224 PMC1914173

[kiad411-B65] Tambasco-Studart M , Titiz O, Raschle T, Forster G, Amrhein N, Fitzpatrick TB. Vitamin B6 biosynthesis in higher plants. Proc Natl Acad Sci U S A 2005:102(38):13687–13692. 10.1073/pnas.0506228102 16157873 PMC1224648

[kiad411-B66] Teixeira ARN , Davies DD. The control of plant glutamate dehydrogenase by pyridoxal-5′-phosphate. Phytochemistry 1974:13(10):2071–2079. 10.1016/0031-9422(74)85005-3

[kiad411-B67] Tilman D , Balzer C, Hill J, Befort BL. Global food demand and the sustainable intensification of agriculture. Proc Natl Acad Sci U S A 2011:108(50):20260–20264. 10.1073/pnas.1116437108 22106295 PMC3250154

[kiad411-B68] Turano FJ , Dashner R, Upadhyaya A, Caldwell CR. Purification of mitochondrial glutamate dehydrogenase from dark-grown soybean seedlings. Plant Physiol. 1996:112(3):1357–1364. 10.1104/pp.112.3.1357 12226451 PMC158064

[kiad411-B69] Wang H , Lu Y, Liu P, Wen W, Zhang J, Ge X, Xia Y. The ammonium/nitrate ratio is an input signal in the temperature-modulated, *SNC1*-mediated and *EDS1*-dependent autoimmunity of *nudt6-2 nudt7*. Plant J. 2013:73(2):262–275. 10.1111/tpj.12032 23004358

[kiad411-B70] Wong HK , Chan HK, Coruzzi GM, Lam HM. Correlation of *ASN2* gene expression with ammonium metabolism in *Arabidopsis*. Plant Physiol. 2004:134(1):332–338. 10.1104/pp.103.033126 14671018 PMC316312

[kiad411-B71] Wulfert S , Krueger S. Phosphoserine aminotransferase1 is part of the phosphorylated pathways for serine biosynthesis and essential for light and sugar-dependent growth promotion. Front Plant Sci. 2018:9:1712. 10.3389/fpls.2018.01712 30515188 PMC6256069

[kiad411-B72] Xu G , Chen W, Song L, Chen Q, Zhang H, Liao H, Zhao G, Lin F, Zhou H, Yu F. FERONIA phosphorylates E3 ubiquitin ligase ATL6 to modulate the stability of 14-3-3 proteins in response to the carbon/nitrogen ratio. J Exp Bot. 2019:70(21):6375–6388. 10.1093/jxb/erz378 31433471 PMC6859809

[kiad411-B73] Yang S , Hua J. A haplotype-specific *Resistance* gene regulated by *BONZAI1* mediates temperature-dependent growth control in *Arabidopsis*. Plant Cell 2004:16(4):1060–1071. 10.1105/tpc.020479 15031411 PMC412877

[kiad411-B74] Zhang Y , Goritschnig S, Dong X, Li X. A gain-of-function mutation in a plant disease resistance gene leads to constitutive activation of downstream signal transduction pathways in *suppressor of npr1-1, constitutive 1*. Plant Cell 2003:15(11):2636–2646. 10.1105/tpc.015842 14576290 PMC280567

[kiad411-B75] Zhang L , Song H, Li B, Wang M, Di D, Lin X, Kronzucker HJ, Shi W, Li G. Induction of *S*-nitrosoglutathione reductase protects root growth from ammonium toxicity by regulating potassium homeostasis in *Arabidopsis* and rice. J Exp Bot. 2021:72(12):4548–4564. 10.1093/jxb/erab140 33772588

[kiad411-B76] Zhao L , Wang Y. Nitrate assay for plant tissues. Bio Protoc. 2017:7(2):e2029. 10.21769/BioProtoc.2029 PMC8376535 34458432

[kiad411-B77] Zimmermann SE , Benstein RM, Flores-Tornero M, Blau S, Anoman AD, Rosa-Téllez S, Gerlich SC, Salem MA, Alseekh S, Kopriva S, et al The phosphorylated pathway of serine biosynthesis links plant growth with nitrogen metabolism. Plant Physiol. 2021:186(3):1487–1506. 10.1093/plphys/kiab167 34624108 PMC8260141

