# Supplementary material for: PYRIDOX(AM)INE 5′-PHOSPHATE OXIDASE3 of Arabidopsis thaliana maintains carbon/nitrogen balance in distinct environmental conditions
Source: Plant Physiol. 2023 Jul 15;193(2):1433–55. doi: 10.1093/plphys/kiad411 (PMC10517258; doi:10.1093/plphys/kiad411)
Supplement: kiad411_Supplementary_Data [file kiad411_supplementary_data.pdf]

(A)

```
PDX3 (At5g49970)    MRNVIRRVTTMTFTFLLQSPPLPISPSPPQFSLSSSPLSKTQRFITPSQGSRLRTLCTKV   60
PNPO-RP (At2g46580) ------------------------------------------------------------   0

PDX3 (At5g49970)    IIPNMQDSGSPPLSYLTQREAAEIDETLMGPLGFSIDQLMELAGLSVAASIAEVYKPEEY   120
PNPO-RP (At2g46580) ------------------------------------------------------------   0

PDX3 (At5g49970)    SRVLAICGPGNNGGDGLVAARHLHHFGYKPFICYPKRTAKPLYTGLVTQLDSLSVPFVSV   180
PNPO-RP (At2g46580) ------------------------------------------------------------   0

PDX3 (At5g49970)    EDLPDDLSKDFDVIVDAMFGFSFHGAPRPPFDDLIRRLVSLQNYEQTLQKHPVIVSVDIP   240
PNPO-RP (At2g46580) ------------------------------------------------------------   0

PDX3 (At5g49970)    SGWHVEEGDHEDGGIKPDMLVSLTAPKLCAKRFRGPHHFLGGRFVPPSVAEKYKLELPSY   300
PNPO-RP (At2g46580) ------------------------------------------------------------   0

PDX3 (At5g49970)    PGTSMCVRIGKPPKVDISAMRVNYVSPELLEEQVETDPTVQFRKWFDE---AV--AAGLR   355
PNPO-RP (At2g46580) ------------------------------------MGTHVAPWKQLLFGAIEANSHLS   23

PDX3 (At5g49970)    ETNAMALSTANKDKKPSSRMVLLKGFDENGFVWF--TNYESKKGSDLSENPSAALLFYWE   413
PNPO-RP (At2g46580) HSSYVQLATIGLNGRPSNRTVVFRGFEENSDRIQINTDLRSRKIEELKHCPFSEMCWYFS   83

PDX3 (At5g49970)    ILNRQVRIEGPVERIPESESEN---------YFHSRPRGSQIGAIVSKQSSVVPGRHVLY   464
PNPO-RP (At2g46580) DTWEQFRINGRIEVIDASNPDQTKLQQREKAWFANSLRSRLIYVC------PTPGSPCNS   137

PDX3 (At5g49970)    DEYEELTKQYSDGSVIPKPKNWGGFRLKPNLFEFWQGQPSRLHDRLQ-YSLQDVNGNPAW   523
PNPO-RP (At2g46580) EQSSQQVK--LDPSSGPVP-EYCLLLLEPEKVDYLNLK---TNQRLFFSSMATGTGEKCW   191

PDX3 (At5g49970)    KIHRLAP 530
PNPO-RP (At2g46580) TSEKVNP 198
```

(B)

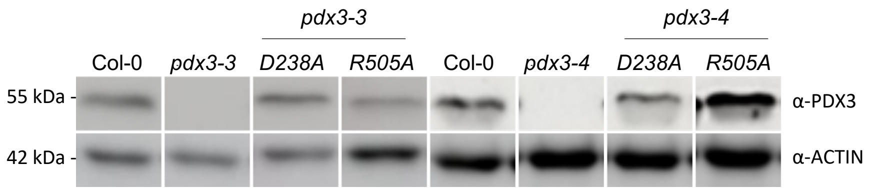

Supplemental Figure S1. Probing the two-domain structure of PDX3. (A) Amino acid sequence alignment of PDX3 (At5g49970, Uniprot: Q9LTX3) and the PDX3 related protein, PNP-RP (At2g46580, Uniprot: Q9ZPY1) described by Marbaix et al 2019. Similar residues are highlighted in gray and highly conserved residues of PDX3s and PNP-RPs as described in Marbaix *et al* 2019 are indicated with a gray arrow. The two PDX3 residues (D238 and R505) mutated in this study are indicated by a bold red font. **(B)** Immunochemical analysis of PDX3 protein levels in wild type (Col-0), *pdx3* and lines expressing either the *PDX3 D238A* or *PDX3 R505A* transgene. The analysis was performed on 14 days old plants grown on soil (unfertilized) under a 16 h photoperiod (120-160 µmol photons m^{-2} s^{-1}) at 22°C and 8 h darkness at 18°C using 20-25 µg of total protein.

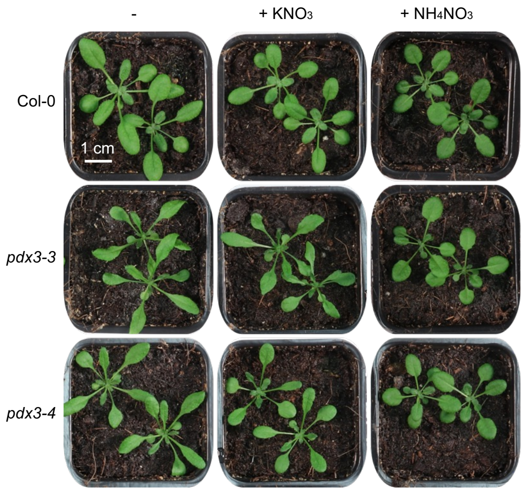

Supplemental Figure S2. Phenotype of rosette leaves of *pdx3* compared to wild type. Photographs of wild type (Col-0) and *pdx3* lines grown on unfertilized (-) and either potassium nitrate (+ KNO_3), ammonium nitrate (+ NH_4NO_3) fertilized soil. The plants are 21 days old and were grown on soil under a 16 h photoperiod (120-160 µmol photons m^{-2} s^{-1}) at 22°C and 8 h darkness at 18°C and were watered either with water alone (-) or a 50 mM solution of the indicated compound every 9-10 days. The scale bar applies to all photographs. Individual images were digitally extracted for comparison.

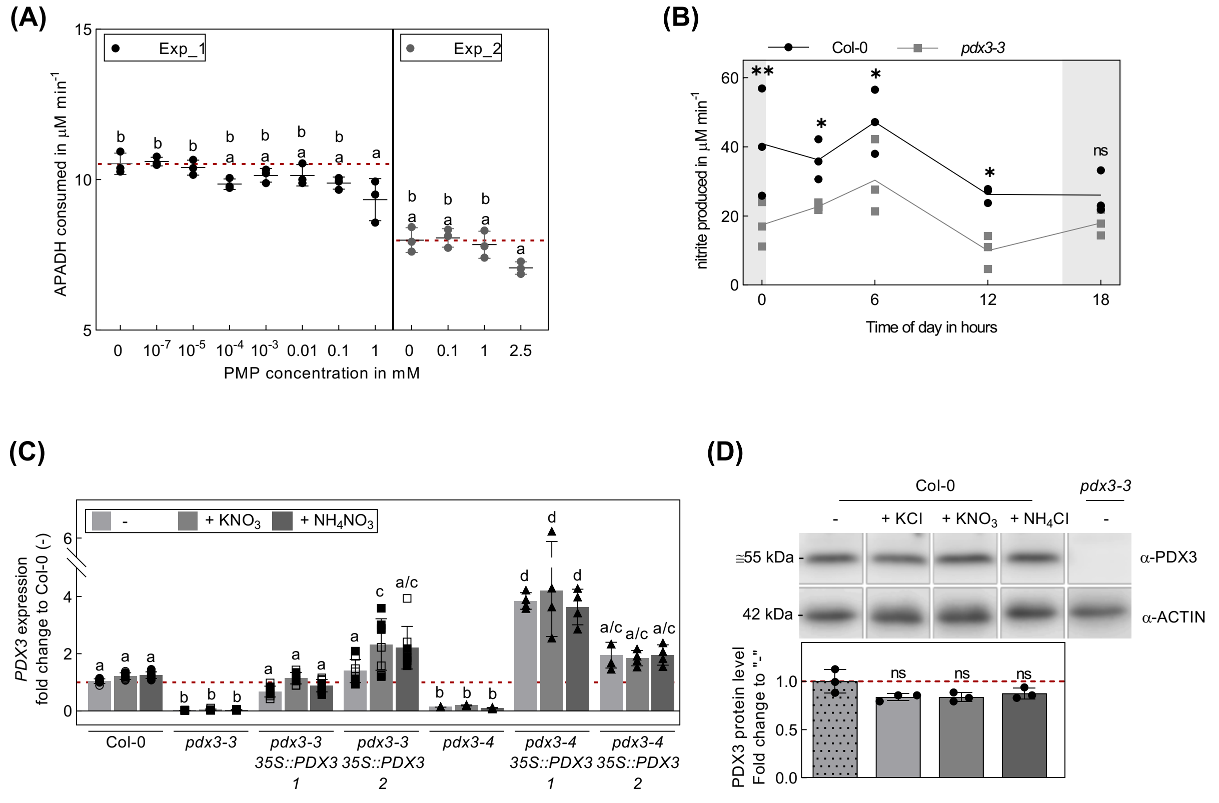

Supplemental Figure S3. Nitrate reductase activity and *PDX3* expression as a function of PMP and N fertilization, respectively. (A) Activity of recombinant nitrate reductase in the presence of PMP, shown as rate of APADH (NADPH substitute) consumption in the presence of 0-2.5 mM PMP at pH 7.5 and 25°C. The data represents the mean ± SD of three technical and two experimental replicates (Exp_1 and Exp_2). Statistical analysis was performed using ordinary one-way ANOVA with Sidak's multiple comparison test (different letters indicate p≤0.05). (B) Nitrate reductase activity in rosette leaves of wild type and *pdx3-3* plants grown on unfertilized soil under a 16 h photoperiod (120-160 µmol photons m^{-2} s^{-1}) at 22°C and 8 h darkness harvested at 0 h (in the dark before the onset of light), 3 h, 6 h, 12 h (all in the light), and 18 h (2 h after onset of darkness). The data represents the mean ± SD of 1 experimental and 3 biological replicates. Statistical analysis was performed using a two-tailed Student's unpaired *t*-test with Col-0 as control (^{ns}p>0.05, *p≤0.05, and **p≤0.005. (C) Relative expression of *PDX3* in *pdx3* and complementing lines compared to wild type (Col-0) grown on unfertilized (-) and either potassium nitrate (+ KNO_3) or ammonium nitrate (+ NH_4NO_3) fertilized soil. Data represents the mean ± SD across 2 experimental replicates, (either open or filled symbols) except for *pdx3-4* and corresponding complementing lines, with 4 biological replicates each. Statistical analysis was performed using an ordinary one-way ANOVA with Tukey's multiple comparisons test for the transcript (different letters indicate p≤0.05). (D) Protein levels of PDX3 in wild type (Col-0) and *pdx3-3* (as a control) grown on unfertilized soil and wild type grown on either potassium chloride (+ KCl), potassium nitrate (+ KNO_3) or ammonium chloride (+ NH_4Cl) fertilized soil. Data represents the mean ± SD of 3 biological replicates. Statistical analysis was performed using a two-tailed Student's unpaired *t*-test using condition (-) as control (^{ns}p>0.05). Plants were grown as in (B) and watered with water alone (-) or a 50 mM solution of the indicated compound every 9-10 days.

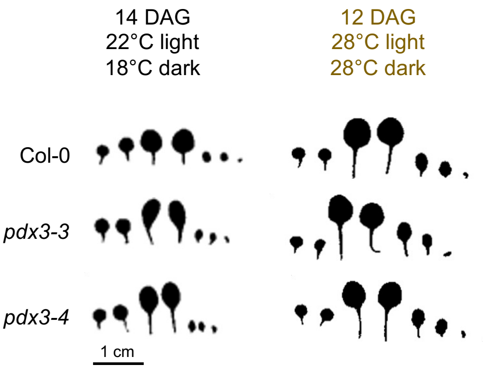

Supplemental Figure S4. Photograph of the leaves of wild type (Col-0) and *pdx3* lines grown up to 14 days after germination (DAG) under the standard temperature of 22°C compared to 12 DAG under 28°C. In these conditions and developmental stage, the number of true leaves (five) is equal. The scale bar applies to all photographs. Individual images were digitally extracted for comparison.

Supplemental Table S1. Oligonucleotides used in this study. Forward (F), reverse (R), left border (LB).

Purpose	Gene	AGI number	Sequence (5′-3′ direction)	Notes
Cloning	*PDX3*	At5g49970	F- CACCTGTGGTGTGGGAAGTCATT	
			R- AGGCCAAACCATCGTCTC	
Site-directed mutagenesis			F- CATTGTCTCTGTGGCTATTCCCTCTGGTTG	D238A
			R- CAACCAGAGGGAATAGCCACAGAGACAATG	
			F- CAGGGACAGCCATCTGCTTTACATGACAGGC	R505A
			R- GCCTGTCATGTAAAGCAGATGGCTGTCCCTG	
Genotyping			F- GCTTTGTCTACAGCGAACAAGG	*pdx3-3*
			R- CGTCGTATAGAACATGCCTGC	
			LB- ATTTTGCCGATTTCGGAAC	T-DNA
			F- ACACACATAGATGTCCCTGGG	*pdx3-4*
			R- AACCCAAACTTGGGTATCACTG	
			LB- ATATTGACCATCATACTCATTGC	T-DNA
	NahG		F- GCCTTAGCACTGGAACTCTG	*NahG*
			R- TCGGTGAACAGCACTTGCAC	
	SID2	At1g74710	F- GGTGCACCAGCTTTTATCGG	*sid2-1*
			R- TGGAGTTGGATGCAGAGCAG	
	NPR1	At1g64280	F- TGCTCTGCAATTGCTCTCCA	*npr1-2*
			R- TGTTGCGGTCTTCACATTGC	
RT-qPCR	*ASN1*	At3g47340	F- GTGGCTTGTTCGACTGCAAAG	
			R- TGAATCACAACTCCTTGACCCA	
	ASN2	At5g65010	F- CGACTGTACCAGGAGGTCCAA	
			R- TTCCATTCTTAGGAAGAGGATCT	
	ATL31	At5g27420	F- ACCGGTGGGCTTTTCTTAG	Pair 1
			R- AACTGACGATGTTCCTTCACC	
			F- TGACCCGTATGCTTACAGCG	Pair 2 (N-supplementation)
			R- CCTGCAGGAGTAACGCTACC	
	GDH2	At5g07440	F- CACTAACGCTCAAACCATGGC	Pair 1
			R- GAACCACCAAGATCAATGGGC	
			F- GACTCGAGCCTTTCACAACATC	Pair 2 (N-supplementation)
			R- AGCGAC TCGGTTAACTCCAAG	
	PR1	At2g14610	F- ACACGTGCAATGGAGTTTGTG	

			R- TTGGCACATCCGAGTCTCACT	
	PDX3	At5g49970	F- GGTAGTGAGTAGAAGTCATGGG	
			R- CGTTTACATGACAGGCTGCA	
	NIA1	At1g77760	F- AGGCTACGCTTATTCTGGAGG	
			R- TGTTCGGTTTCTCCTGGTGG	
	NIA2	At1g37130	F- GTTTCATTGGTGGCCGGATG	
			R- TACCACCAACCTTCTTCGTCG	
	ACT2	At3g18780	F- TTGTTCCAGCCCTCGTTTGT	
			R- CCTGGACCTGCCTCATCATACT	
	UBC21	At5g25760	F- TAGCATTGATGGCTCATCCTGA	
			R- TTGTGCCATTGAATTGAACCC	
